# Antioxidants in Animal Nutrition: UHPLC-ESI-Q*q*TOF Analysis and Effects on In Vitro Rumen Fermentation of Oak Leaf Extracts

**DOI:** 10.3390/antiox11122366

**Published:** 2022-11-29

**Authors:** Marialuisa Formato, Alessandro Vastolo, Simona Piccolella, Serena Calabrò, Monica Isabella Cutrignelli, Christian Zidorn, Severina Pacifico

**Affiliations:** 1 Department of Environmental, Biological and Pharmaceutical Sciences and Technologies, University of Campania ‘Luigi Vanvitelli’, Via Vivaldi 43, 81100 Caserta, Italy; 2 Department of Veterinary Medicine and Animal Production, University of Naples Federico II, Via Federico Delpino 1, 80137 Napoli, Italy; 3 Pharmazeutisches Institut, Abteilung Pharmazeutische Biologie, Christian-Albrechts-Universität zu Kiel, Gutenbergstraße 76, 24118 Kiel, Germany

**Keywords:** *Quercus robur* L., oak leaves, UHPLC-ESI-Q*q*TOF HR-MS analysis, flavonoids, condensed tannins (CTs), volatile fatty acids, in vitro fermentation

## Abstract

The genus *Quercus* supplies a large amount of residual material (e.g., bark, acorns, leaves, wood), the valorization of which can favor a supply of antioxidant polyphenols to be used in the pharmaceutical, nutraceutical, or cosmeceutical sector. The recovery of specialized metabolites could also benefit livestock feeding, so much so that polyphenols have gained attention as rumen fermentation modifiers and for mitigating the oxidative imbalance to which farm animals are subject. In this context, leaves of *Quercus robur* L. from Northern Germany were of interest and the alcoholic extract obtained underwent an untargeted profiling by means of ultra-high-performance liquid chromatography/high-resolution tandem mass spectrometry (UHPLC-HRMS/MS) techniques. As triterpenes and fatty acids occurred, the alcoholic extract fractionation pointed out the obtainment of a polyphenol fraction, broadly constituted by coumaroyl flavonol glycosides and condensed tannins. Total phenol, flavonoid and condensed tannins content assays, as well as antiradical (DPPH^●^ and ABTS^+●^) and reducing activity (PFRAP) were carried out on the alcoholic extract and its fractions. When the effects on rumen liquor was evaluated in vitro in terms of changes in fermentation characteristics, it was observed that oak leaf extract and its fractions promoted an increase in total volatile fatty acids and differently modulated the relative content of each fatty acid.

## 1. Introduction

The genus *Quercus* (Fagaceae family) consists of trees that are distributed worldwide, with an estimated 450 species with marked differences in morphological shape and chemical composition. *Quercus robur* L., known as pedunculate or English oak, together with *Quercus petraea* (Matt.) Liebl., the sessile or durmast oak, is a common broadleaved tree species in Europe, widespread also in Asia and North America. In Europe, *Quercus robur* L. reaches northwards to southern Norway and Sweden and southwards to the northern part of the Iberian Peninsula, Southern Italy, the Balkan Peninsula and Turkey [1]. The *Quercus* species produces a fruit, the acorn, which, together with bark and leaves, has been used in folk medicine to treat various diseases [2,3]. The ancient use of acorns in human and animal diet could be due to its diversity in macronutrients (carbohydrates, proteins and fatty acids) and antioxidant compounds. In fact, phenolic acids (e.g., gallic acid, ellagic acid and their derivatives), flavonoids (e.g., quercetin, catechin, naringin) and tannins [4,5] were previously identified in acorn extracts. However, diversely from other nut plants, such as chestnut (*Castanea sativa* Mill.), walnut (*Juglans regia* L.), hazelnut (*Corylus avellana* L.), pistachio (*Pistacia vera* L.), peanut (*Arachis hypogaea* L.) and others, *Quercus* spp. fruits lack consideration in actual human nutrition [6].

Currently, the European wood industry pays attention to *Quercus* ssp., as *Q. robur* L., *Q. petraea* L. and *Q. alba* L. produce high-quality hardwood for construction and furniture manufacture [1,7]. The oak wood serves also for manufacturing oak barrels for wine maturation and, mainly as part of wood processing, for colouring and preserving wood against fungal decay [7,8].

Forest residues are from oak wood processing, but unutilized oak barks and leaves also contain bioactive compounds (flavonoids, saponins and hydrolysable and condensed tannins [9]) of interest for pharmaceuticals, food, or cosmetics applications [3,10,11]. It is noteworthy that barks of *Q. robur*, *Q. petraea* and *Q. pubescens* are listed in the official Pharmacopoeia Database [12] and that decoctions from *Q. robur* and *Q. petraea* barks were recognized as exhibiting anti-inflammatory, antibacterial and antihemorrhagic activities [2]. In addition, the use of bark powder of *Q. robur* L. is reported for diarrhoea prophylaxis in cattle, horses, pigs, sheep and chickens [13]. Moreover, oak leaves have found application for treating gastrointestinal, inflammatory, chronic skin diseases or urinary infections [14,15], or for preparing infusions with medicinal or nutritional purposes [2]. Furthermore, together with twigs, *Quercus* ssp. leaves are harvested for use during feed shortages and applied in animal nutrition, being grazed by ruminants [16]. Indeed, oak leaves could represent innovative feed ingredients to achieve sustainable animal production, jointly maintaining or even improving animal health, performance and product quality, since the banning of feed antibiotics by the EU in 2006 [17,18,19,20]. They contain high levels of hydrolysable and condensed tannins [9,15,21,22], which can act as rumen modifiers, modulating the diversity and activity of rumen microorganisms, or nutrients’ digestibility. In this regard, it has been widely shown that tannins, as well as flavonoids, can selectively reduce the growth of bacteria involved in carbohydrate fermentation, such as *Butyrivibrio fibrisolvens*, *Streptococcus bovis* and *Ruminobacter amylophilus*, also impacting on methanogenesis [23]. Moreover, it was found that oak tannins supplemented in the diet of lactating Holstein cows reduced urinary nitrogen excretion and increased the α-linolenic acid levels in milk [24].

Oak polyphenols could be further exploited as natural antioxidants to counteract inflammatory disease onset in animal farming. Antioxidants in animal feeding, are a strategy to ensure a correct animals’ redox status, to improve animal performance and to further improve the quality of products such as milk and meat, with benefits for humans [25]. Based on these considerations, herein, *Q. robur* L. leaves, collected in the same area of the previously investigated *Fagus sylvatica* L. [26] and *Castanea sativa* Mill. leaves, were of interest and the alcoholic extract by maceration was first investigated through an untargeted UHPLC-HRMS/MS approach. Furthermore, the extract underwent fractionation and all the obtained fractions, differently enriched in bio-actives, were preliminarily screened for their total phenol (TPC), flavonoid (TFC) and condensed tannins (TCT) as well as for their antiradical and reducing activity by means of DPPH^●^ and ABTS^●+^ tests and by ferricyanide FRAP assay. The further chemical investigation of the two organic fractions was carried out by means of UV-Vis spectroscopy and UHPLC-HR MS/MS. Furthermore, all the differently chemically constituted fractions were tested to evaluate their effects on in vitro ruminal fermentation (cumulative gas production; organic matter degradability; fermentation kinetics; and end products, i.e., pH, volatile fatty acids, branched-chain fatty acid proportion and acetate/propionate ratio).

## 2. Materials and Methods

### 2.1. Plant Collection, Fractionation and Evaluation of Leaf Chemical Composition

The leaves of *Quercus robur* L. were collected in August 2021 in the Botanical Garden of Kiel University (Kiel, Germany, N 54°20′52″, E 10°06′58″, 20 m a.m.s.l., Google Earth). The leaves underwent extraction and fractionation as reported by Formato et al. [26] with some modifications. The leaves were first lyophilized (ScanVAC CoolSafe, Labogene, Brigachtal, Germany) and pulverized by a rotating knife homogenizer (IKA^®^ MF 10 basic, Staufen, Germany). Dried leaves underwent classical maceration overnight, for five days, using methanol as extractive solvent. The drug/solvent ratio was 1:4 (mg drug: mL solvent). The alcoholic extract (Qr/1/1), obtained with a yield equal to 21.1 % (210.9 g), was then dissolved in a biphasic solution CHCl_3_:MeOH:H_2_O (13:7:6, *v*:*v*:*v*) and discontinuous liquid-liquid extraction (LLE) was performed for three cycles providing an organic fraction (Qr/2/1; 28.2% of Qr/1/1) and a hydroalcoholic one (Qr/2/2). This latter underwent liquid-liquid extraction (LLE) using water (H_2_O) and butanol (BuOH). Thus, the organic fraction Qr/3/2 was obtained with a yield equal to 32.7%. Oak leaves were also analyzed according to the procedures of the Association of Official Agricultural Chemists [27] to determine dry matter (DM), ether extract (EE), crude protein (CP) and ash. The fiber fractions (Neutral Detergent Fiber on organic matter basis, NDFom, Acid Detergent Fiber on organic matter basis, ADFom, and Acid Detergent Lignin, ADL) were also determined according to Van Soest et al. [28].

### 2.2. UHPLC-HRMS and MS/MS Parameters and UV-Vis Analyses

The alcoholic extract, Qr/1/1 and the fractions therefrom were first analyzed by UV-Vis spectrophotometry in the range 200–800 nm using an Agilent Cary 100 UV/Vis Spectrophotometer (Agilent; Santa Clara, CA, USA). The three samples (10 mg/mL) were profiled by a NEXERA UHPLC system (Shimadzu; Tokyo, Japan) equipped with Luna^®^ Omega C-18 column (1.6-µm particle size, 50 × 2.1 mm i.d.) and 2.0 µL of each sample were injected. The separation was achieved using a binary solution: (A) H_2_O and (B) CH_3_CN both with 0.1 % formic acid (HCOOH). A linear gradient was used in which the percentage of solvent B increased as follows: 0–2 min, 2% B; 2–12 min, 2% → 15% B; 12–18 min, 15% → 35% B; 18–28 min, 35% → 75% B; 28–30 min, 75% → 95% B, 30–32 min, 95% B; 32.01–34.00 min, column re-equilibration. The flow rate was set at 500 µL/min. The AB SCIEX TripleTOF^®^ 4600 (AB Sciex, Concord, ON, Canada) system was equipped with a DuoSpray^TM^ ion source, with the ESI probe used for MS investigations in negative ionization mode and the APCI probe used for fully automatic mass calibration, using the Calibrant Delivery System (CDS). CDS injects a calibration solution matching the polarity of ionization and calibrates the mass axis of the analyzer in all scan functions (MS or MS/MS). A full-scan time-of-flight (TOF) survey (dwell time 250 ms, 100–1500 Da) and eight IDA MS/MS scans (dwell time 100 ms, 80–1300 Da) were acquired, using the following parameters: curtain gas (CUR) 35 psi, nebulizer (GS1) and heated (GS2) gases 60 psi, ion spray voltage (ISVF) 4500 V, ion source temperature (TEM) 600 °C and declustering potential (DP) −80 V. The collision energy (CE) applied was −40 V, with a collision energy spread (CES) of 5 V. The instrument was controlled by Analyst^®^ TF 1.7 software (AB Sciex, Concord, ON, Canada, 2016), while data processing was carried out using PeakView^®^ software version 2.2 (AB Sciex, Concord, ON, Canada, 2016).

### 2.3. Radical Scavenging Capacity: DPPH and ABTS Tests

The antioxidant capability of the investigated oak leaf extract and its fractions was assessed by assays based on ABTS (2,2′-azinobis-(3-ethylbenzothiazolin-6-sulfonic acid)) radical cation and DPPH (2,2′-diphenyl-1-picrylhydrazyl) radical. The samples were tested at 200, 100, 50, 25, 12.5, 6.25 and 3.125 µg/mL (final concentrations). Trolox (2, 4, 8, 16 and 32 µM) was used as the positive standard. All recorded activities were compared to a blank sample, arranged in parallel.

The samples were added to the DPPH^●^ methanol solution (9.4 × 10^−5^ M); the mixtures were stirred for 15 min and the absorption was read at 517 nm by a Wallac Victor3 spectrophotometer (PerkinElmer, Waltham, MA, USA), in reference to a blank.

The investigated samples were added to ABTS^●+^ solution. This latter was obtained by diluting with PBS (phosphate-buffered saline; pH 7.4) until an absorbance equal to 0.7 at 734 nm was obtained, the ABTS^●+^ solution generated by the reaction between (2,2′-azinobis-(3-ethylbenzothiazolin-6-sulfonic acid); 7 mM) and potassium persulfate (K_2_S_2_O_8_; 2.45 mM), in the dark for 12 h. The mixtures reacted, under stirring, for 6 min, and after that the absorbance was measured using a Wallac Victor3 spectrophotometer referring to a blank [29].

The results were expressed in terms of the percentage reduction of the initial radical adsorption by the tested samples, applying the formula ((A_Blank_ − A_sample_)/A_Blank_) × 100 [30], where A_Blank_ is blank absorption and A_sample_ is sample absorption. The ID_50_ and TEAC values were calculated.

### 2.4. Determination of Potassium Ferricyanide Reducing Power (PFRAP)

The potassium ferricyanide reducing power (PFRAP) assay was also performed to estimate the Fe(III) reducing power of Qr/1/1 alcoholic extract and its fractions (200, 100, 50, 25, 12.5, 6.25 and 3.125 µg/mL, final concentrations). The absorbance was measured at 700 nm using a Wallac Victor3 spectrophotometer [31]. The increase in absorbance referring to the blank was calculated. Trolox (4, 8, 16 and 32 µM) was the positive standard.

### 2.5. Determination of Total Phenol Content

The total phenol content (TPC) was determined according to the Folin-Ciocalteau procedure [29]. Samples (0.25 mg and 0.125 mg) were mixed with 2.25 mL of Na_2_CO_3_ (2.25 mL; 7.5% *w*/*v*) and 0.25 mL of Folin-Ciocalteu reagent. The tubes were mixed and allowed to stand for 3 h at room temperature (T = 25 °C), in the dark. The absorbance was read at 765 nm using a Synergy spectrophotometer (Biotek, Winooski, VT, USA). Data were expressed as milligrams of gallic acid equivalents (GAEs) per g of extract. To this purpose, a gallic acid calibration curve (y=0.0247x−0.0063; *R*^2^ = 0.9998) was built up in the range 0.78–25 µg/mL (final concentration levels).

### 2.6. Determination of Total Flavonoid Content

The total flavonoid content (TFC) was determined adding NaNO_2_ (5%, *w*/*v*; 0.3 mL) to the samples (1 mg and 2 mg), which were firstly solubilized into 5 mL of distillate water. After 10 min, AlCl_3_ solution (10%, *w*/*v*; 0.6 mL) was added. The reaction was carried out for 6 min. Then, NaOH aqueous solution (1.0 M; 2.0 mL) was added and the mixture was further diluted to 10 mL with distillate water. The absorbance was read at 510 nm against the blank (water) using a Synergy spectrophotometer. The flavonoid content was expressed as milligrams of quercetin equivalents (QUEs) per g of extract [26], using a quercetin calibration curve (y=0.0243x−0.0038; *R*^2^ = 0.9978). This latter was built up in the range 0.78–50 µg/mL (final concentration levels).

### 2.7. Determination of Total Condensed Tannins

The total condensed tannin (TCT) content was determined according to Butanol-HCl method [32], with some modifications. The samples (0.1 mL) were dissolved in 3 mL of the Butanol-HCl reagent (95:5, *v*:*v*; HCl concentrated 37%) and 0.1 mL of the ferric reagent (2% *w*/*v* in 2 N HCl) was added. The tubes were mixed and put in a heating block adjusted at 97 °C for 60 min. The absorbance was read at 550 nm against the blank (water) using a Synergy spectrophotometer (Biotek, Winooski, VT, USA). Data were expressed as milligrams of cyanin equivalents (CYEs) per g of extract. To this purpose, a cyanin calibration curve (y=0.0339x−0.1202; *R*^2^ = 0.9946) was built up in the range 1.95–62.5 µg/mL (final concentration levels).

### 2.8. In Vitro Fermentation

Oak Qr/1/1 extract and its Qr/2/1 and Qr/3/2 fractions were incubated at 0 (control), 50 and 200 mg dose levels with the control diet (1.0055 ± 0.0024 g). This latter was composed by corn silage, oat hay and concentrate (NDF: 44.2% and CP: 13.7%).

All the substrates were incubated with rumen fluid (10 mL) at 39 °C in hermetically closed 120 mL serum flasks under anaerobic conditions. Buffered medium (75 mL) and reducing agent (4 mL) were added [33]. Three replications for each extract and dosage (3 × 3 × 2)) were performed. The rumen fluid was collected at a slaughterhouse authorized according to EU legislation [34], from six healthy young bulls (*Bos taurus*). All procedures concerning animals were accepted by the Ethical Animal Care and Use Committee of the University of Napoli Federico II (Prot. 2019/0013729 of 8 February 2019). Then, the collected rumen liquor was transferred to the laboratory of the Department of Veterinary Medicine and Animal Production of the University of Napoli Federico II. There, it was pooled flushing with CO_2_, filtered through a cheesecloth and added to the flasks. The gas produced during the 120 h of incubation was reordered using a manual pressure transducer (Cole and Palmer Instrument Co, Vernon Hills, IL, USA) and related to incubated OM (OMCV, mL/g). At the end of incubation (120 h), the pH of the fermentation liquor was measured using a pH meter (ThermoOrion 720 A+, Fort Collins, CO, USA). Subsequently, the organic matter degradability (OMD, %) was assessed by weight differences of the incubated OM and the undegraded filtered (sintered glass crucibles; Schott Duran, Mainz, Germany, porosity # 2) residue burned at 550 °C for 3 h [35]. The in vitro fermentation analysis is outlined in Figure S1.

### 2.9. Fermentation End Products Assessment

To assess the volatile fatty acids (VFAs, mmol/g) production, the fermentation liquor of each bottle after 120 h of incubation was first cooled at 4 °C and then centrifuged at 12,000× *g* for 10 min (Universal 32R centrifuge, Hettich FurnTech Division DIY, Melle-Neuenkirchen, Germany) [36]. The supernatant (1 mL) was mixed with oxalic acid (1 mL: 0.06 mol). The VFA composition was evaluated by gas chromatography (ThermoQuest 8000top Italia SpA, Rodano, Milan, Italy) equipped with a fused silica capillary column (30 m, 0.25 mm ID, 0.25 μm film thickness) by means of an external standard solution composed of pure acetic, propionic, butyric, iso-butyric, valeric and iso-valeric acids. Branched-chain fatty (BCFA) acids percentages were calculated as follows:

((iso-butyric acid + iso-valeric acid)/tVFAs) × 100.

### 2.10. Data Processing and Statistical Analysis

Colorimetric tests were carried out performing three replicate measurements for three samples (*n* = 3) of the extracts (in total, 3 × 3 measurements). All data were expressed as mean ± SD values.

To estimate the fermentation kinetic parameters, the gas production profiles were fitted to the sigmoidal model [37]:

G=A/1+BtC.

where G is the total gas produced (mL/g of incubated OM) at time t (h); A is the asymptotic gas production (mL/g), B is the time at which one-half of A is reached (h) and C is the curve switch. The maximum fermentation rate (R_max_, mL/h) and the time at which it occurs (T_max_, h) were determined using model parameters [38]:

Rmax=A×CB× B × TmaxB−1(1+CB×Tmax−B)2. Tmax=C×( B−1B+1)1B

Tmax=C×(B−1B+1)1B

Statistical analyses were performed by ANOVA (JMP^®^, Version 14 SW, SAS Institute Inc., Cary, NC, USA, 1989–2019). Post-Hoc Dunnett test has been performed to observe the differences between control and experimental diets. The significance level was verified at *p* < 0.05, *p* < 0.01 and *p* < 0.001. Statistical comparison Shapiro-Wilk test for normally distributed data has been performed. In addition, principal component analysis (PCA), as well as Violin Plot and Heatmap, were carried out by Origin2015 and GraphPad Prism 8.4.2., respectively.

## 3. Results

### 3.1. Oak Leaf Nutritional Value for Livestock Feed

**Figure 1:**
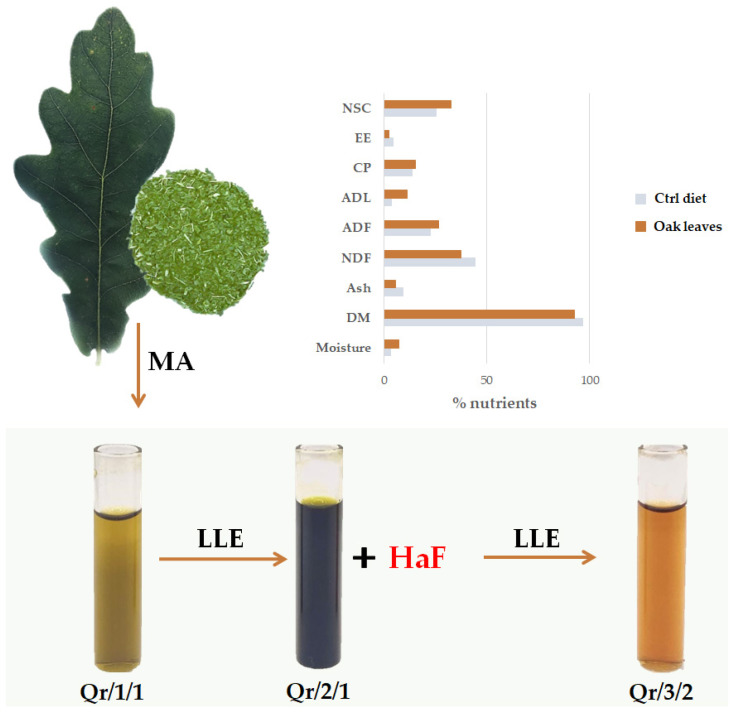
*Quercus robur* L. leaf chemical composition parameters expressed as % of DM and fractionation scheme to achieve bioactive fractions. DM: dry matter; NDF: neutral detergent fiber; ADF: acid detergent fiber; ADL: acid detergent lignin; CP: crude protein; EE: ether extract; NSC: no structural carbohydrates; MA: maceration; LLE: liquid–liquid extraction; HaF: hydroalcoholic fraction.

The quality of oak leaves as animal diet ingredients was evaluated in terms of dry matter (DM), ash, neutral (NDF) and acid (ADF) detersed fiber, acid detersed lignin (ADL), ether extract (EE), no structural carbohydrates (NSC) and crude protein (CP), compared to a standard control diet. Data acquired are reported in Figure 1 and expressed on dry matter basis. NDF and ADF were 37.3 and 26.4% DM, respectively, whereas the ADL was equal to 11.1% DM. The crude protein content was equal to 15.3%, overcoming the minimum level of crude protein for grazing species [39].

### 3.2. The Fractionation of an Alcoholic Oak Leaf Extract Showed up Antioxidant Polyphenols

**Figure 2:**
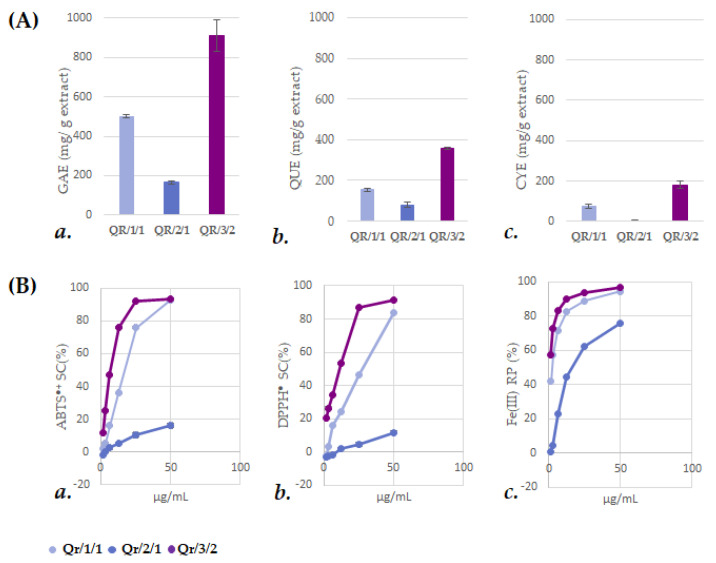
(**A**) a. Total phenolic content (TPC), expressed as mg of gallic acid equivalents (GAEs) per g of extract; b. total flavonoid content (TFC), expressed as mg of quercetin equivalents (QUEs) per g of extract; c. total condensed tannin content (TCT), expressed as mg of cyanin equivalents (CYEs) per g of extract. Values reported are the mean ± SD of three independent measurements. (**B**) a. Scavenging capability (SC%) vs. 2,2′-azino-bis(3-ethylbenzothiazoline)-6-sulfonic acid (ABTS) radical cation; b. scavenging capability (SC%) vs. 2,2-diphenyl-1-picrylhydrazy (DPPH) radical; c. Fe(III) reducing power (RP%) of *Q. robur* extract and organic fractions therefrom. Values reported are the mean ± SD of three independent measurements.

In order to hypothesize the recovery of specialized metabolites, leaves underwent alcoholic maceration to obtain Qr/1/1 extract (Figure 1). The latter was subjected to fractionation to achieve a non-polar fraction Qr/2/1 and a hydroalcoholic one, which further provided the alcoholic fraction Qr/3/2. Qr/1/1 extract and the organic fractions therefrom (Qr/2/1 and Qr/3/2) were preliminarily investigated for their total content of phenols (TPC), flavonoids (TFC) and condensed tannins (TCT) (Figure 2). The parental extract (Qr/1/1) showed a discrete phenolic and flavonoid content equal to 501.7 ± 5.5 gallic acid equivalents (GAEs) and 154.1 ± 8.7 quercetin equivalents (QUEs) per g of extract, respectively.

Following fractionation, Qr/3/2 was a polyphenol-enriched fraction with values of TPC and TFC equal to 909.5 ± 80.6 gallic acid equivalents (GAEs) and 359.3 ± 6.1 quercetin equivalents (QUEs) per g of extract, respectively. Moreover, its total condensed tannin content was equal to 181.8 ± 18.9 cyanin equivalents (CYEs) per g of extract. In this regard, the CTs characteristic reaction, which is based on the oxidative cleavage of the interflavan bond providing anthocyanidins, led us to use cyanine as standard for calibration curve. Anti-radical (DPPH^●^ and ABTS^+●^ tests) and Fe(III) reducing power (PFRAP) assessment corroborated previous data. The fraction Qr/2/1 exhibited the lowest values of Trolox Equivalent Antioxidant Capacity (TEAC) in line with TPC, TFC and TCT data. In contrast, Qr/3/2 markedly scavenged both ABTS^●+^ and DPPH^●^, with relative TEAC values equal to 0.63 (ID_50_ = 6.61 ± 0.03 µg/mL) and 0.72 (ID_50_ = 8.24 ± 0.39 µg/mL), respectively. In particular, it was observed that Qr/3/2 was able to almost convert DPPH radical completely in its reduced form at 25 µg/mL dose level, which scavenged the radical by 87%. Moreover, Qr/3/2 effectively reduced ferric ions at the lowest doses, exhibiting TEAC and ID_50_ values equal to 3.88 and 0.41 ± 0.10 µg/mL, respectively (Figure 2B(a–c)).

### 3.3. Chemical Insights into the Qr/1/1 Extract and Its Qr/2/1 and Qr/3/2 Fractions

**Figure 3:**
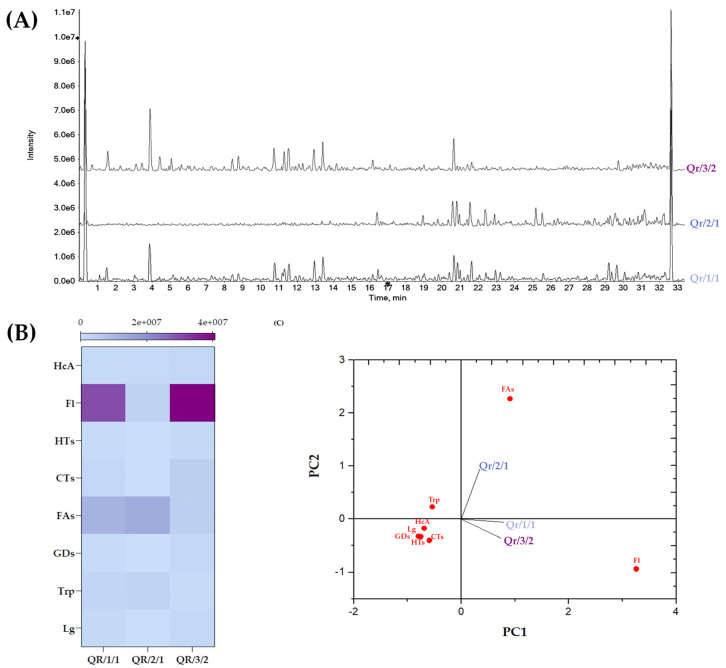
(**A**) Total ion current (TIC) chromatograms of Qr/1/1 extract, Qr/2/1 fraction and Qr/3/2 fraction. (**B**) Heatmap of the relative content of each extract/fraction in derivatives of gallic acids (GDs), hydroxycinnamic acid (HcA), lignans (Lg) as well as flavonoids (Fl), hydrolysable (HTs) and condensed tannins (CTs), fatty acids (FAs) and triterpenes (Trp), is shown. Principal component analysis is on the left, based on different classes of identified compounds with PC1% variance = 68.5 and PC2% variance = 31.5.

UV-Vis spectra of Qr/1/1 fractions highlighted that fraction Qr/2/1 was enriched mainly with lipophilic compounds, while tannins and flavonoids mainly constituted Qr/3/2 (Figure S2). In fact, Qr/3/2 UV-Vis spectrum showed bands at 370 and 285 nm, in line with flavonoids and condensed tannins electronic transitions. The UV-Vis absorption bands at 670 and 415 nm of Qr/2/1 suggested its content in chlorophylls and carotenoids, whereas those at 234 and 205 nm were attributable to fatty acids, their oxidative derivatives and triterpenoids. To obtain insights into the chemistry of Qr/1/1 extract and fractions derived, UHPLC-HR MS/MS analyses were performed (Figure 3, Table S1).

#### 3.3.1. Tannins in Oak Leaf, beyond Other Low-Molecular Weight Compounds

Quinic acid (**1**), gallic acid (**3**) and two isomers of gallic acid hexoside ([M − H]^−^ at *m*/*z* 331.0676(57); (**2**,**4**) were identified in the alcoholic extract and Qr/3/2 fraction, together with hydrolysable (HTs) and condensed (CTs) tannins, whose structures are schematized in Table 1.

Among hydrolysable tannins, ellagitannins (**9**, **23**, **32** and **37**) and gallo-tannins (**25**,**27** and **30**) turned out together with ellagic acid (**39**) ([M − H]^−^ at *m*/*z* 300.9996; C_14_H_6_O_8_), although in an amount lower than procyanidins. Compound **32** with [M − 2H]^2−^ at *m*/*z* 467.0375, in accordance with molecular formula C_34_H_32_O_31_, was tentatively galloyl-bis-HHDP-glucose. Compound **9**, which has one less unit of gallic acid, has been identified as bis-HHDP-hexose. The loss of HHDP-group (302 Da) provided the ion [M − H-HHDP]^−^ at *m*/*z* 481.0625 while the ellagic acid, produced by the loss of the HHDP group, is displayed by the fragment ion at *m*/*z* 300.9975. Metabolites **23** ([M − H]^−^ at *m*/*z* 785.0870) and **37** ([M − 2H]^2−^ at *m*/*z* 468.0438) were tentatively digalloyl-HHDP-hexose and trigalloyl-HHDP-glucose, respectively. In the TOF-MS/MS spectra of compound **23**, the typical losses of galloyl, gallic acid and HHDP group occurred, within fragment ions at *m*/*z* 633.0690, 615.0645, 483.0784. Digalloyl deoxyhexose and trigalloyl hexose were recognized (**25** and **30**). In both TOF-MS/MS spectra, galloyl moiety was identified, based on the characteristic loss of 152.01 Da and the relative fragment ions at *m*/*z* 169.0127 and 169.0131. TOF-MS/MS spectrum of the compound **25** showed also the fragment ion at *m*/*z* 125.0252 and its radical ion at *m*/*z* 124.0151.

Compound **27** was galloyl-methylgalloyl hexose, in which methyl-gallic acid occurrence was unravelled thanks to the fragment ion at *m*/*z* 183.0288 (Figure S3).

Condensed tannins (CTs) were only in Qr/3/2, in a relative amount higher than in Qr/1/1. This class of compounds, also known as procyanidins (PAs), are flavan-3-ol oligomers and polymers of catechin and (epi)catechin, that yield anthocyanidins upon oxidative acid depolymerization reactions. The flavan-3-ol monomer units are sometimes esterified with gallic acid to form 3-*O*-gallate derivatives. Unlike procyanidins, prodelphinidin structures are made of gallocatechin. Type B procyanidins are dimers resulting from interflavanoid linkages with C4 → C8 or C4 → C6. Procyanidins A-type lacks two hydrogens compared to the B-type ones due to C–O bond between the C5 and C7 carbons of the upper units, in addition to the C-C interflavan linkage. Trimers or C-type procyanidins consist of three flavan-3-ol units linked by two C4 → C8 interflavan bonds [40]. Using high-energy collision-induced dissociation with negative ion tandem mass spectrometry, cleavages between monomeric subunits formed three types of class characteristic and structurally significant product ions consisting of quinone methide (QM), heterocyclic ring fission (HRF) and retro-Diels-Alder (RDA) fragment ions [41,42,43]. Thus, highly diagnostic fragment ions allowed CTs to be putatively identified. HRF and RDA fragmentations provided information about the hydroxylation of the B-rings and bonds between monomeric units while quinone methide (QM) fragmentation defined the monomeric units.

Three metabolites (**5**, **7** and **22**) with [M − H]^−^ at *m*/*z* 593.13, in accordance with molecular formula C_30_H_26_O_14_, were tentatively identified as (epi)gallocatechin-(epi)catechin isomers (Figure S4). TOF-MS/MS spectra showed the characteristic fragment ions at *m*/*z* 425.08, from which the consequent water loss provided ion at *m*/*z* 407.07, 467.09 and 289.07. These fragment ions derived by HRF, RDA and QM reaction, respectively. The presence of (epi)gallocatechin monomeric unit was further confirmed by fragment ions at *m*/*z* 305.06 and 303.05 as well as by the neutral loss of 126.03 Da (pyrogallol moiety), which in turn gave the ion at *m*/*z* 177.01. Instead, compound **6** ([M − H]^−^ at *m*/*z* 609.1265 C_30_H_26_O_14_) was tentatively identified as (epi)gallocatechin dimer (Figure S5). The ion at *m*/*z* 305.0656, corresponding to the monomeric unit, was from QM (quinone methide) fission and interflavanic bond cleavage. In addition, the RDA derived B-ring loss, identified by ions at *m*/*z* 441.0825 and 423.0716, further confirmed the presence of a pyrogallol moiety, which was further confirmed by loss of 126.03 Da (pyrogallol moiety). Three (epi)catechin dimer isomers (**12**, **14** and **31**) (Figure S6), with deprotonated molecular ions at *m*/*z* 577.13, were tentatively identified. The TOF-MS/MS spectra showed the characteristic fragment ions of HRF (*m*/*z* 451.10), RDA (*m*/*z* 425.08) and QM (*m*/*z* 289.07).

**Figure 4:**
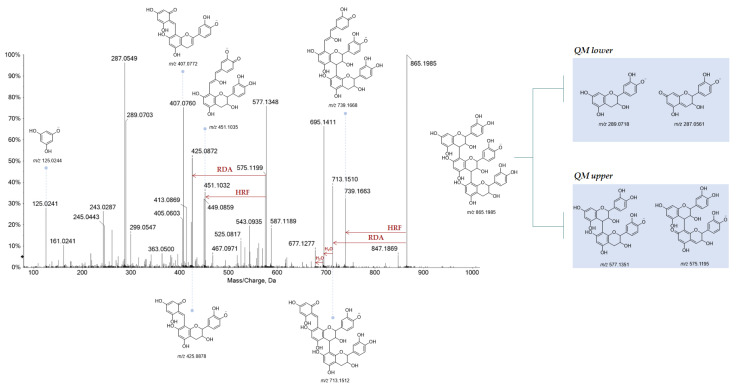
TOF-MS/MS spectrum of compound **19**. The chemical structure of each product ion is depicted and its theoretical *m*/*z* ratio is indicated below.

**Figure 5:**
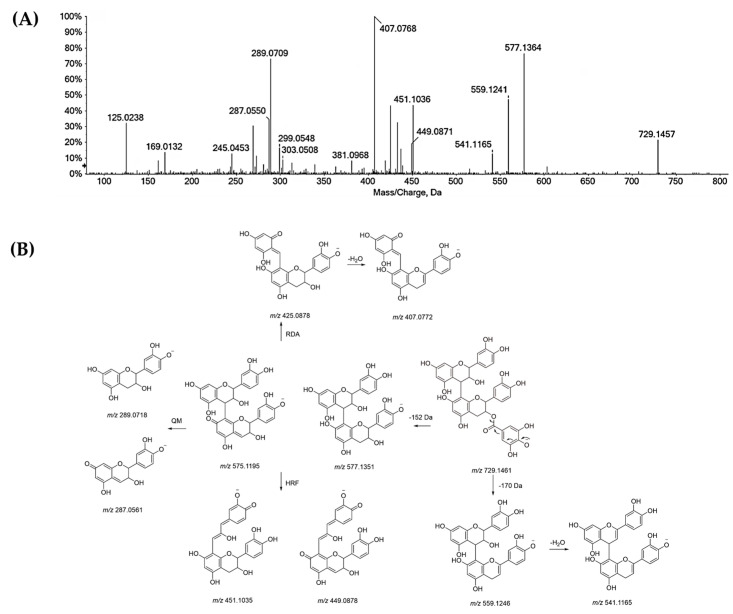
(**A**) TOF-MS/MS spectrum of compound **29** and (**B**) its putative pattern of fragmentation.

Trimers of (epi)catechin were also recognized (**18**, **19** and **28**). TOF-MS/MS spectra (Figure 4) showed fragment ions at *m*/*z* 577.13 and 289.07 from QM fragmentation with their conjugated derivatives at *m*/*z* 575.12 and 287.05, respectively. The fragment ions at *m*/*z* 739.16 and 451.10 were obtained by HRF fragmentation from ions at *m*/*z* 865.19 and 577.13, respectively. TOF-MS/MS spectrum (Figure 5) of the compound **29** ([M − H]^−^ at *m*/*z* 729.1457) suggested (epi)catechin dimer 3-*O*-gallate occurrence. The 152 Da and 170 Da losses provided the fragment ions at *m*/*z* 577.1364 and 559.1241, respectively. These losses could be attributable to the presence of gallate moiety. Beyond these fragment ions, TOF-MS/MS spectrum displayed the typical ions derived by QM, HRF and RDA fragmentation.

Among hydroxycinnamoyl based compounds, 3-*O* and 5-*O*-*p*-coumaroyl quinic acids (**10**, **11**, **24**, **26**) were detected as well as a feruloyl quinic acid (**17**) [44]. Moreover, compounds **96** and **97** were putatively identified as eicosyl *p*-coumarate and docosyl caffeate, respectively. These compounds were also in fraction Qr/2/1 due to their esterification with 20 or 22 carbons alkyl chains, which confer high apolarity to cinnamoyl core. Eicosyl *p*-coumarate, as well as other alkyl coumarates and ferulates, was also identified in *Ipomoea carnea* subsp. *fistulosa* (Convolvulaceae family) [45] and in leaf and root cattails (*Typha domingensis* Pers. and *Typha latifolia* L.) [46]. Eicosanyl and docosyl caffeate were isolated from *Glycyrrhiza glabra* and they exhibited potent elastase inhibitory activity [47]. Losses of the alkyl chains, 280.31 Da (eicosyl moiety) and 308.34 Da (docosyl moiety), provided ions at *m*/*z* 163.0388 and 179.0394, diagnostic for *p*-coumaric and caffeic acid, respectively.

Finally, compounds **44** and **45** were identified as neolignan-*O*-deoxyhexoside isomers, as previously reported in *F. sylvatica* alcoholic leaf extract [31].

#### 3.3.2. Flavonoids in Oak Leaf

Beyond flavonols, which constituted the main flavonoidic component and whose structures are schematized in Table 2, some catechins and one flavanone were putatively identified. The deprotonated molecular ions at *m*/*z* 289.0709(05) for compounds **13** and **16**, eluting at two different retention time, were in accordance with catechin and epicatechin diasteroisomers [43]. The CO_2_ loss, with consecutive A-ring cleavage, provided fragment ions at *m*/*z* 245.0809(8); characteristic ions formed by HRF reaction at *m*/*z* 125.0239 and RDA at *m*/*z* 137.0243(35) and 151.0393(86) were also observed (Figure S7). Compound **8** was tentatively identified as (epi)gallocatechin. Beyond fragment ions common to all catechins, TOF-MS/MS spectra displayed diagnostic fragment ions of B-ring at *m*/*z* 137.0235 and 167.0444, which are by RDA reaction and 139.0483, derived by benzofuran-forming fission (BFF).

Quercetin (**54**, [M − H]^−^ ion at *m*/*z* 301.0356), kaempferol (**63**, [M − H]^−^ ion at *m*/*z* 285.0393), isorhamnetin (**69**, [M − H]^−^ at *m*/*z* 315.0495) and myricetin were found as the most abundant flavonol aglycone cores. A flavanone, such as eriodictyol 7-*O*-hexoside (**20**, [M − H]^−^ ion at *m*/*z* 449.1089), was also tentatively identified; the molecular deprotonated ion provided an ion at *m*/*z* 287.0541 following the neutral loss of a hexose moiety and the fragment ion at *m*/*z* 259.0601 by neutral loss of CO (28 Da) and hexose moiety (162.05 Da). Myricetin 3-*O*-hexosides (**34** and **36**) and myricetin 3-*O*-pentosylhexoside (**33**) were putatively identified. In fact, the loss of 162.05 Da (dehydrated hexose) and 294.09 Da (dehydrated hexose + pentose) suggested the glyconic moiety identity, whereas fragment ions at *m*/*z* 317.02 was attributable to myricetin together with its aglycone radical anion at *m*/*z* 316.02. The abundance of this latter allowed us to hypothesize the C-3 linkage of sugar moieties.

Different mono- and diglycosidic derivatives of quercetin (**35**, **38**, **40**–**43** and **46**) and isorhamnetin (**50**–**52**) were also identified. TOF-MS/MS spectra of compounds showed the classical neutral losses due to dehydrated hexose, pentose (132.04 Da), hexuronic acid (176.03 Da), di-hexose (324.10 Da) and pentosylhexose, providing the [aglycone-H]^−^ and [aglycone-H]^−●^ ions at *m*/*z* 301.03/300.02 and 315.05/314.04, for quercetin and isorhamnetin, respectively. For isorhamnetin, a further loss of 15 Da, providing fragment ions at *m*/*z* 300.02 and 299.01, supported the structural hypothesis. Moreover, the different [aglycone-H]^−^/[aglycone-H]^−●^ ratio in the TOF-MS^2^ spectra of compounds led to hypothesizing the sugar position. Among kaempferol mono- and diglycosidic derivatives, kaempferol hexosides **47** and **48**, kaempferol pentosylhexoside **49** and kaempferol (acetyl)-hexosides **53** and **58**, also detected in Qr/2/1 fraction, were tentatively identified.

**Figure 6:**
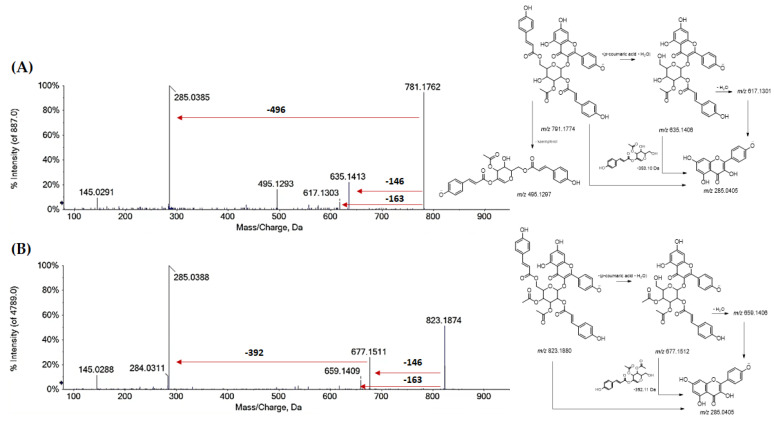
TOF-MS/MS spectra of compound **79** (**A**) and **82** (**B**). Proposed fragmentation pattern for each deprotonated molecular ion is reported. The chemical structure of each fragment ion is depicted and its theoretical *m*/*z* ratio is indicated below.

Different quercetin, isorhamnetin and kaempferol acylated derivatives were in the alcoholic extract and its Qr/3/2 fraction. Compound **56** ([M − H]^−^ ion at *m*/*z* 741.1704) and metabolites **60** and **61** ([M − H]^−^ ion at *m*/*z* 609.1254(77)), were tentatively identified as quercetin *p*-coumaroyl-pentosylhexoside and quercetin *p*-coumaroyl hexoside, respectively. Moreover, isorhamnetin *p*-coumaroyl hexoside (**64**) and isorhamnetin di-*p*-coumaroyl hexoside (**76**), as well as some acyl derivatives of kaempferol, were also putatively identified in fraction Qr/2/1. Mono- and di-*p*-coumaroyl kaempferol glycosides, with one or more acetyl units, were detected (**62**, **68**, **72**–**75**, **78**, **79**, **81** and **82**) and they seemed to be the most abundant among the acyl derivatives. Coumaroyl flavonols were tentatively identified for the first time in oak leaves. Representatively, TOF-MS/MS spectra of compounds **79** and **82** are reported in Figure 6. While hydroxycinnamoyl glycosides of quercetin, kaempferol, isorhamnetin were putatively identified through the neutral losses of 308.09 Da (*p*-coumaroylhexose-H_2_O) and 454.13 Da (di-*p*-coumaroylhexose-H_2_O), when a di-*p*-coumaroyl residue occurred together with an acetyl one, the deprotonated molecular ion appeared to undergo the loss of dehydrated *p*-coumaric acid (−146 Da) or *p*-coumaric acid (−163 Da) to provide kaempferol *p*-coumaroyl(mono- or di-)acetylhexoside. The neutral loss of *p*-coumaroylhexose-H_2_O, both mono- or diacetylated, was favored so much so that kaempferol anion was formed as the peak with highest intensity.

#### 3.3.3. Fatty Acids in Oak Leaf

**Figure 7:**
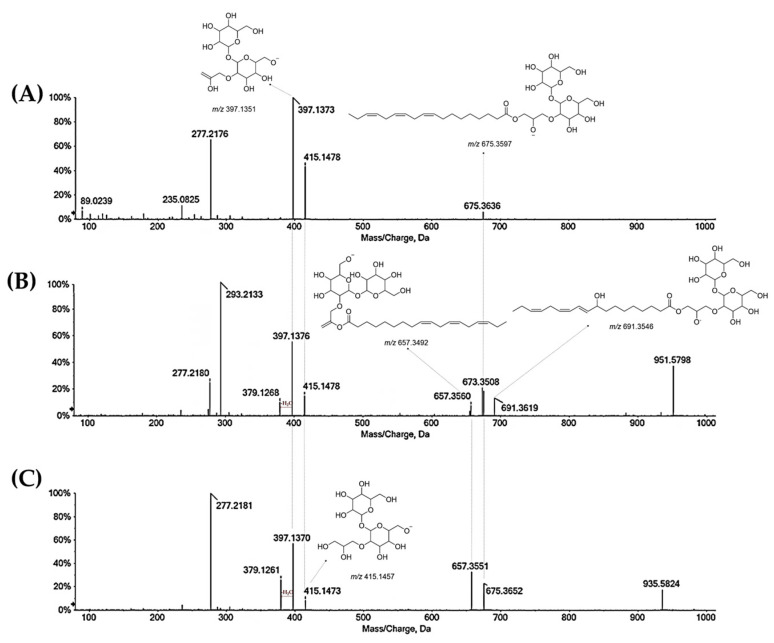
TOF-MS/MS spectra of compound **83** (**A**), **93** (**B**) and **95** (**C**). The chemical structure of each product ion is depicted and its theoretical *m*/*z* ratio is indicated below.

Beyond flavonoids and procyanidins, the alcoholic extract and its main Qr/2/1 fraction contained fatty acids and terpenoids (Table S1). Compounds **55** and **57** ([M − H]^−^ at *m*/*z* 389.1808(25)) were putatively identified as hydroxy-dihydrojasmonic acid hexoside. Among fatty acids were dodecanedioic acid (**67**, [M − H]^−^ at *m*/*z* 227.1267), likely traumatic acid, 9,12,13-trihidroxy-10,15 octadecadienoic acid (**70**, [M − H]^−^ at *m*/*z* 327.2172), 9,12,13-trihydroxy-10-ocadecenoic acid (**71**, [M − H]^−^ at *m*/*z* 329.2320) and 9-hydroxy-10,12,15-octadecatrienoic acid (**84**, [M − H]^−^ at *m*/*z* 293.2113). TOF-MS/MS of compound **84**, beyond common neutral loss of water, showed the ions at *m*/*z* 171.1017 and 121.1034, diagnostic for identifying hydroxyl group position. Polyunsaturated fatty acids **90** ([M − H]^−^ at *m*/*z* 277.2179; n:3) and **92** ([M − H]^−^ at *m*/*z* 279.2332; n:6) were recognised, together with monounsaturated oleic acid at *m*/*z* 281.2488 (**94**), digalactosylmonoacylglycerol (DGMG) (**83**), monogalactosylmonoglycerol (MGMG) (**86**), digalactosyldiacylglycerols (DGDG) (**93** and **95**) and glycerophospholipids (**85**, **88** and **89**). TOF-MS/MS spectra of compounds **83**, **93** and **95** showed diagnostic signals arising from the polar head: the ion at *m*/*z* 415.14 (two galactose residues linked to the *sn*-3 position of the glycerol backbone) and two ions at *m*/*z* 397.13 e 379.12, derived by sequential losses of one (for compound **83**) or two water molecules (for compounds **88** and **89**), most likely arising from glycerol and/or galactosyl groups (Figure 7). The most favoured loss as FA is known to occur for the acyl chain located in the *sn*-1 position of glycerol [48,49]. The identification of acyl components was confirmed by ions at *m*/*z* 691.36, 675.36 and 657.35 as well as fragment ions at *m*/*z* 277.2176(81) (linolenic acid) and 293.2123 (hydroxylinolenic acid). Compounds **85** and **88** were tentatively *lyso*-PA (18:3) and *lyso*-PA (18:2), respectively, for low intensities of their linked fatty acids [50]. Instead, compound **89** was likely oleoyl-diglycerol-phosphate. In fact, its TOF-MS/MS spectrum showed diagnostic ions at *m*/*z* 281.2475 (oleanolic acid) and 152.99 (dehydro-phosphoglycerol).

Furthermore, fraction Qr/2/1 accounted for a substantial triterpene aglycones. According to literature data, bartogenic acid and its derivatives were isolated from oak heartwood [51] and wood [52], as well as different tetrahydroxyolean-12-ene-24,28-dioic acid derivatives [53]. However, little information is available in the literature on the isolation and identification of these metabolites in *Q. robur* L. leaves. Bartogenic acid (**77**, [M − H]^−^ at *m*/*z* 517.3171) and different pentacyclic triterpenes (**59**, **80**, **87** and **91**) (e.g., ursolic, oleanolic, corsolic or maslinic acid) were identified. Compounds **65** and **66**, with [M − H]^−^ at *m*/*z* 679.3720(18) and molecular formula C_36_H_56_O_12_, could be bartogenic acid hexoside isomers. The TOF-MS/MS spectra displayed the ions at *m*/*z* 559.3264 and 517.3149, derived by X^0,2^ cross-link cleavage of hexose and dehydrated hexose loss, respectively. The consecutive losses of H_2_O and CO_2_ from fragment ion at *m*/*z* 517.3149 (bartogenic acid) gave fragment ions at *m*/*z* 499.3042 and 455.3157, respectively.

### 3.4. Effects of Oak Leaf Alcoholic Extract and Its Fractions on In Vitro Rumen Fermentation

**Figure 8:**
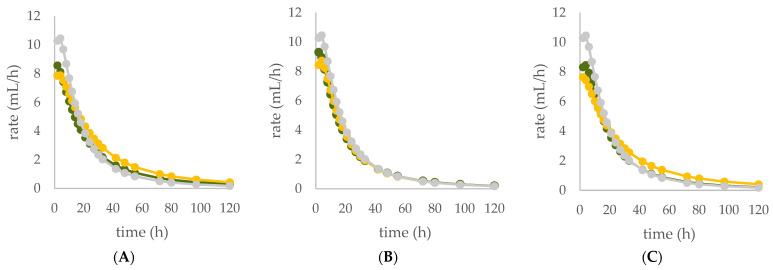
In vitro fermentation rate over time of *Q. robur* Qr/1/1 extract (**A**) and its fractions Qr/2/1 (**B**) and Qr/3/2 (**C**) at the 50-mg (●) and 200-mg (●) dose levels and control diet (●).

The diet supplemented with Qr/1/1 and its derived fractions impacted in vitro gas production at ruminal level and fermentation rate over time (Table 3; Figure 8A–C). After 120 h of incubation, all oak based diet supplemented with 50 mg of extract/fraction reduced significantly (*p* < 0.001), compared to the control diet, the organic matter degradability (OMD). Contrariwise, at 200 mg dose level Qr/1/1 did not modify OMD, whereas Qr/2/1 and Qr/3/2 were able to decrease it by 3.7 and 12.6%, respectively. It was reported that the increase of condensed tannins led to a reduction of OMD as well as of total gas production after only 24 h of incubation [54]. In addition, oak leaves exhibited OMD reduction after 96 h of incubation [55], whilst showed a value equal to 56.22 ± 0.68% after 48 h of incubation [56]. Similarly, OMCV after 120 h showed values lower than control diet, mainly for all oak-based diet at 50 mg (*p* < 0.001). In particular, the lipophilic fraction Qr/2/1, at both tested dose levels, exhibited the lowest values (*p* < 0.001) for OMCV. Furthermore, only the Qr/1/1 and Qr/2/1 at 50 mg dose level as well as Qr/3/2-200 mg showed the lowest T_max_ value (*p* < 0.05). Contrariwise, all oak samples at 200 mg reduced mainly R_max_ (*p* < 0.001), which did not appear to have been affected by Qr/2/1-50 mg treatment. Gas volume is a valid indicator of substrates fermented in VFAs and an index of potential digestibility in the rumen. Thus, the curve of gas production of all extracts at 50 mg exhibited a decrease compared to control diet as well as Qr/2/1-200 mg dose level. The kinetic profile obtained incubating Qr/1/1 and Qr/3/2 at 200 mg displayed an increase of the trend compared to control diet after 50 and 80 h, respectively. The fermentation rate obtained incubating all the samples at 50 mg dose level appeared to be lower than that of control diet, whereas Qr/1/1 and Qr/3/2 at 200 mg displayed an increase of fermentation rate after 20 h of incubation.

### 3.5. Influence of Oak Leaf Alcoholic Extract and Its Fractions on Fermentation End Products

Data acquired in terms of pH and concentration of the fermentation end products as total VFA, BCFA and A/P ratio, recorded after 120 h of incubation, are listed in Table 4(A) with relative ViolinPlots (Table 4(B)). In vitro fermentation end products according to the single volatile fatty acids were reported as proportion (%) of the single volatile fatty acids towards the total volatile fatty acids content, expressed as mmol/L (Table 5).

The rumen pH was not modified by Qr/1/1, whereas it was significantly increased by Qr/2/1 (*p* < 0.01) and Qr/3/2 (*p* < 0.05) treatment. In particular, Qr/2/1 at 200 mg dose level displayed a value equal to 6.37.

The total volatile fatty acids (tVFA) increased and the observed effect for all the tested samples appeared to be dose-dependent (Table 4), except for Qr/2/1. In fact, the dose level massively impacted the total volatile fatty acids amount because with the dose of 50 mg, an increase equal to 35.5 and 23.7% was observed for Qr/1/1 and Qr/3/2, respectively. Qr/3/2-200 mg increased by 46.9% followed by Qr/1/1-50 mg which increased 1.4-fold the VFA. Instead, Qr/2/1, at both the dose levels, led to an increase of VFAs between 20.0 and 30.0%. Taking into account branched chain fatty acids (BCFAs), it was found that they significantly decreased (*p* < 0.001) by about 60.0% following all treatments. The most impacting effect was by Qr/2/1 at 200 mg dose. Similarly, the A:P ratio appeared to decrease significantly at all dose levels, although an increase in polyphenols, as it is in Qr/1/1 and Qr/3/2 fractions, provided a less consistent decrease.

Among the main volatile fatty acids involved in the ruminal methane regulation, acetate and propionate decreased and increased, respectively. Qr/2/1 and Qr/3/2, regardless of dose, reduced by 17–18% the acetate production after 120 h of incubation, which displayed the lowest decrease with Qr/1/1, mainly at dosage of 200 mg, for which no significant variation occurred. Propionate positively increased at all extracts, mainly at Qr/2/1-200 mg. However, the effect seemed to be negatively impacted by polyphenol increase, as observed for A/P ratio. In fact, Qr/1/1 and Qr/3/2 at 50 mg increased propionate by 39.1 and 65.6%, respectively, but at 200 mg alcoholic extract and its fraction led to an increase equal to 27.8 and 54.5%, respectively. Butyrate appeared to massively augment (*p* < 0.01), by all treatment without a significant variation intra-dose. Qr/3/2 showed the highest increase with a mean value equal to 54.6%. Significant increase was displayed also for Qr/1/1 (*p* < 0.05) and Qr/2/1 (*p* < 0.01) at percentages ranging from 26 to 46, respectively. On the contrary, valerate appeared to decrease by more than 50% in all treatments except for Qr/2/1-200 mg, which reduced by 41.7%. In the same way, iso-valerate and iso-butyrate appeared to decrease at all treated samples. For iso-valerate, the greatest decreases were observed by Qr/2/1-200 (68.0%) and Qr/3/2-50 mg (62.8%), whereas Qr/3/2-200 mg exhibited the lowest decrease (30.3%). On the contrary, Qr/3/2 treatment at both the doses affected iso-butyrate content, while Qr/2/1-50 mg dose level reduced it by only 3.0%.

The dendrograms from data of total VFAs related to the diet at the dose level of 50 and 200 mg are in Figure S8A. Both the dendrograms showed three clusters: the first group included tVFA and acetic acid, the second accounted in propionic and butyric acid and the third included two subgroups with valeric acid, A/P, BCFA (IIIa) and iso-valeric acid with iso-butyric acid (IIIb). Principal component analysis (PCA) (Figure S8B) at 50 mg showed the positive correlation of tVFA and acetic acid with alcoholic fraction (Qr/1/1), which showed a discrete amount of TFC (501.6 ± 5.5 gallic equivalents (GAEs) per g of extract), TPC (154.1 ± 8.7 quercetin equivalents (QUEs) per g of extract) and TCT (73.5 ± 10.1 cyanin equivalents (CYEs) per g of extract). At 200 mg, tVFA was positively correlated not only to Qr/1/1 but also to enriched polyphenol fraction (Qr/3/2), which showed the highest amount of TFC (*r* = 0.847), TPC (*r* = 0.937) and TCT (*r* = 0.910). An opposite trend was shown for acetic acid, which was positively correlated to the apolar fraction, as well as propionic and butyric acids. Other volatile fatty acids showed similar distribution at both strengths.

## 4. Discussion

Plant extract screening studies globally have become an important research field for the development of alternative feed additives to ensure animal health and manipulate rumen ecology by reducing greenhouse gases. Among the plant secondary metabolites, polyphenols have received considerable attention for human and animal nutrition due to their wide range of biological activities. In this regard, several studies have been carried out on the effects of phenolic compounds on rumen fermentation and microbiota. Moreover, polyphenols can affect in mitigating oxidative stress in ruminants under moderate stress conditions, which are frequent in intensive livestock farming. However, information on extract phytochemical properties, in terms of effects on rumen enzyme activities, microbiota, rumen fermentation process or anti-inflammatory mechanisms is fragmented and sometimes discordant. Since tree foliage is a source of protein, energy and minerals for herbivorous animals, the evaluation of the nutritional value and chemical composition of *Quercus robur* L. leaves is a key step to verify if they cover the nutritional needs of ruminants.

Oak leaves and twigs are often grazed by animals or used as livestock fodder, although toxicosis episodes occurred when the intake of tannins was in high doses [16]. The quality of the forage mainly depends on the amount and quality of fiber, while, according to previous data, it was found that structural carbohydrate contents in oak leaves are lower than in other plant parts such as cup, hull, kernel and whole-fruit, whilst they exhibit higher contents of dry matter and crude protein [55]. As oak leaves are known to contain specialized metabolites, in order to clarify the impact of extraction/fractionation processes in their recovery and the relation chemical composition/activity at rumen level, an alcoholic extract was first prepared by maceration and fractionation was employed to provide the polyphenol fraction Qr/3/2. Assessing anti-radical efficacy, it was proved that oak leaf polyphenol fraction markedly scavenged both radical probes, while its ferric ions reducing activity was slightly higher than that of Qr/1/1. Polyphenol compounds are main actors in antioxidant efficacy, as detected in test tube assays, as they are able to transfer both hydrogen atom and single electron to radical species. This occurs also for oak leaf polyphenols. Indeed, although the relative content of the intrinsic polyphenol heritage of a plant organ of an extract/fraction is mostly affected by extractive techniques employed, previous reports are in line with the preparation of an hydroalcoholic extract (ethanol:water, 4:6, *v*:*v*) from oak leaves, able to exert scavenging activity towards several oxygen (ROS) and nitrogen (RNS) reactive species [57]. Antioxidant plant extract could slow down the overproduction of reactive oxygen species (ROS), which, together with other proinflammatory stimuli, such as LPS and cytokines, commonly leads livestock to oxidative stress, making it more susceptible to various diseases (e.g., mastitis, metritis, placenta retention, infertility, SARA) [17]. Flavonoids, hydrolysable tannins and procyanidins, displaying antioxidant efficacy [10,14,15,22,58,59] as well as antimicrobial [10,60,61] and antitopoisomerase activities [60], were found to be constituent of different *Quercus* ssp. organs (e.g., leaves, root, bark). In particular, a *Q. robur* L. methanolic extract from the aerial plant parts exhibited antimicrobial activity against *Escherichia coli* and *Staphylococcus aureus* [62], whereas an hydroalcoholic leaf extract exhibited an important hepato- and gastro-protective activity [59]. Moreover, different leaf extracts of *Q. robur* showed a discrete inhibitory effect against α-amylase and *β*-glucosidase [15]. To obtain insights into the relative content in specialized metabolites and their effects on rumen fermentation rate and gas production, UHPLC-HR MS/MS untargeted analyses were carried out (Figure 3A). Alcoholic oak leaf extract accounted of flavonoid mono-, di- and acyl-glycosides, condensed tannins and beyond fatty acids and terpene compounds. In addition, ellagitannins were also identified, in relatively low amount. This could be due to the seasonality of tannins, so much so that the biosynthesis of proanthocyanidins increases during summer season in despite of hydrolyzable tannins [63,64]. In fact, *Q. robur* leaves were collected in summer in the wooded site of the city of Kiel (Northern Germany), together with *Fagus sylvatica* [26] and *Castanea sativa* leaves, in a research program aimed at evaluating the recovery of foliage for feed purposes from the three species. As climatic factors can influence the qualitative-quantitative composition of metabolites in different organs of a plant as well as its nutrients [63,65], a common collection time of the leaves of the three species was considered to avoid seasonal variability of specialized metabolites. When fractionation was carried out, tannins and flavonoids appeared to constitute Qr/3/2. This was confirmed by the UV-Vis spectrum of the fraction (Figure S2), whose bands at 370 and 285 nm, were in line with flavonoids and condensed tannins electronic transitions. On the contrary, fraction Qr/2/1 was enriched mainly of lipophilic compounds. This partitioning was confirmed by the following UHPLC-HR MS/MS analyses. It deserves attention that the relative content of flavonoids in Qr/3/2 fraction was almost 10-fold that of CTs and that flavonoid component accounted by the 40% in coumaroyl flavonol glycosides. Although these compounds were never reported as oak constituents, their occurrence were, for example, from leaves of *Camellia sinensis* [43], male flowers of *Gingko biloba* [66] and burs of *Castanea crenata* [67]. Coumaroylation was found to be realized in plant cell walls and its status can be used as an indicator of the type of tissue in a plant. Structure–activity relationship carried out on astragalin and its *p*-coumaroyl derivative, by using DPPH and ABTS methods, as well as Fe-binding assay, showed that the presence of the *p*-coumaroyl moiety enhanced the efficiency of the HAT-based pathways [68]. Kaempferol derivatives, both acetylated and acylated by means of coumaroyl moieties, were recently isolated from leaves of *Pasania dodoniifolia* [69]. Coumaroyl flavonol glycosides have never been investigated, especially in s livestock feeding scenario. In this context, plant extracts, often generically referred to as polyphenol mixtures, are of interest and their ability to act as modifiers of bacterial activities in rumen broadly fits with environmentally friendly and safe food production systems. However, since this is a relatively new research field, the literature data regarding the beneficial effects of polyphenols are often inconsistent. Unquestionably, the class of tannins is the one most paid attention to. It is widely reported that excessive and prolonged intake of tannin-rich leaves can induce toxicity due to their astringent property which reduces feed intake and, consequently, animal performance [66]. Furthermore, condensed tannins have been observed to limit the bioavailability of minerals (Al, Ca, Co, Cu, Fe, Mg, Mn, P and Zn), forming a CT-metal ion complex, stable over a wide pH range [70]. Hydrolysable and condensed tannins appeared also to increase the digestive utilization of dietary protein, due to their ability to bind proteins in the rumen, preventing their excessive microbial degradation. Thus, to date, various strategies have been applied to improve the utilisation of tannins in animal husbandry. Beyond the control of daily intake dosage, ruminants exhibit some adaptation mechanisms such as the shift in rumen microbial population towards microbes that can degrade tannins or production of proline rich tannin-binding proteins in saliva. In this way a reduction of their intrinsic toxicity may occur with consequent improvement of the utilization of tannins enriched feeds. It is generally assumed that a concentration of condensed tannins up to 50 mg/g/d is beneficial for ruminants, but this largely depends on the chemical nature of the tannin source [71]. Nevertheless, literature data on the effects of oak pedunculate tannins leaf, alone or in combination with flavonoids, are limited, unlike those on chestnut, acacia and quebracho extracts. It has been shown that oak polyphenol enriched-diet, mainly when supplemented with Qr/3/2, significantly reduced organic matter digestibility. This effect could be due, based on actual literature data, to CTs therein, but the important presence of flavonol compounds could not be ignored. In fact, CTs are known for their ability to bind proteins and to play an important role also in carbohydrate metabolism, because of their ability to complex microbial enzymes, altering microbial populations involved in fiber digestion [72]. In this context, it was found that CTs inhibit the ability of *Fibrobacter succinogenes* in digesting fiber through inactivation of extracellular enzymes and interference with the adhesion to cellulose fibers [73]. Flavonoids, such as myricetin, kaempferol and catechin, were observed to reduce the population of rumen microbes, also affecting xylanase and β -glucosidase activities purine content and the efficiency of microbial protein synthesis [74,75]. The double-edge sword of polyphenols, especially flavonoids, is likely to underlie the dose-dependent modulation of the fermentation kinetic by oak leaf extract/fraction-enriched diet, with effect more pronounced when the 50 mg dose was added. In this case also, Kamalak et al. [76], investigating nutritive value of oak leaves, highlighted the correlation between crude protein content and tannins and gas production. Moreover, oak leaves were also able to reduce the methane emission and ammonia nitrogen with negative effect on organic matter digestibility and metabolized energy [77]. The addition of the partially purified fractions from oak leaves, Qr/2/1 and Qr/3/2, resulted in an increase of rumen pH. This finding is not in line with data from the screening of CTs extract from *Leucaena leucocephala* [54], while Hassanat and Benchaar [78] proved a significant increase in rumen pH after only 24 h of incubation with different hydrolysable (chestnut and valonea) and condensed tannins extracts (acacia and quebracho). The effect of a flavonoid mixture on rumen pH was also established and flavonoid supplementation was proven to improve rumen fermentation, while reducing the incidence of rumen acidosis [79]. The possibility that CTs were not the only players in the exercise of the recorded activities was further suggested by analysing data on the relative concentration of the end products. In fact, a marked increase in total VFAs was observed, while a decrease was previously observed from CT extract of *Leucaena leucocephala* Lam., which was able to reduce VFAs, propionate and butyrate after 24 h of incubation [54], as well as from a CT extract of both acacia and quebracho [78]. This literature evidence allowed us to hypothesize that the significant effects herein observed on end products could be attributable to the high flavonoid content [26]. The massive BCFAs decrease by Qr/2/1 at 200 mg dose and the less consistent decrease by Qr/1/1 and Qr/3/2 suggested that supplementation could differently lead to disruption of microbial digestion involved in protein metabolism. Indeed, peptides can be degraded by rumen peptidases into amino acids (AAs), which can be incorporated into microbial proteins or further deaminated into branched volatile fatty acids, CO_2_ and ammonia. If energy is available, the AAs undergo transamination or used directly for microbial protein synthesis. However, if energy is limited, the AAs deaminate and their carbon skeleton undergoes fermentation into VFAs [80,81]. Therefore, a reduction of protein degradation in the rumen is associated with a lower production of ammonia and a greater flow of non-ammoniacal nitrogen to the duodenum and a higher amount of branched fatty acids. In this context, it has been observed that the proanthocyanidin fractions from the leaves of some species of *Ficus* or *Anogeissus pendula* and *Eugenia jambolana*, mainly composed of (epi)catechin, (epi)gallocatechin and their 4-phloroglucinol adducts, decrease the glutamic oxaloacetic ruminal and pyruvic transaminases as well as rumen R-Cellulase enzymes with consequent effects on the use of fibers and proteins [82,83]. Dietary condensed or hydrolysable tannins, supplied at adequate concentrations, are agreed to reduce in vitro concentrations of rumen NH_3_ and branched-chain volatile fatty acids. However, differences in results between studies may be related to variation in feedstuffs quality, CT concentration, or affinity of CT sources for carbohydrates and proteins.

Qr/3/2 was able to increase propionate levels, significantly reducing A:P ratio. This is in accordance with data from a *Q. cortex* L. methanol extract (trited at 0.96 mg CTs/1 g extract), which increased propionate, while reducing acetate, whose activity enhanced when it was combined with *Vaccinium vitis idaea* L. leaves (trited at 37.1 mg CTs/1 g extract) [84]. Contrariwise, heartwood extract of *Q. robur*, composed of ellagitannins, did not change any parameters in terms of VFAs, acetate, propionate, butyrate and A/P, except when in combination with hop pellet [85]. These literature data further support, in our case, the capability of flavonoids, together with proanthocyanidins, to influence rumen fermentation parameters. The butyrate increases and the effect recorded on single branched FAs also were according to data from studies in which phenolic compounds from oak (*Q. robur* with *Q. petraea*) were observed to modulate rumen fermentation without causing any negative effects on DM digestibility. Moreover, commercial oenological tannin extracts, at dose of 20 g/kg diet DM, positively increased PUFA, 18:3 (n-3), 18:2 (n-6) and trans-11 18:1 followed by decrease of trans-10 18:1 and 18:0 rumen concentrations [86]. It is noteworthy to know that fatty acids and triterpenes in Qr/2/1 fraction could be responsive for the recorded modification of some fermentation parameters. In this regard, Jalc et al. [87] demonstrated an influence of C-18 unsaturated fatty acids (oleic, linoleic and α-linolenic acids) on rumen fermentation in terms of total VFAs, acetate, propionate, butyrate and methane, as well as on fatty acid metabolism. This finding could address the optimization of feeding formula in which all the constituents of the oak leaf could play a different “active” role.

## 5. Conclusions

The pedunculated oak leaves have been shown to be a natural source of structurally different metabolites, capable of exerting valuable effects on rumen fermentation during 120 h of in vitro incubation under anaerobiosis condition.

Data acquired underline that fractionation strategies must be employed to enhance the efficacy of each class of specialized metabolites, which coexist like an orchestra within the oak leaf organ. In fact, the constitution of polyphenol fraction could benefit in terms of antioxidant efficacy, also impacting fermentation end products at rumen level. The flavonol diversity of oak leaves was extraordinarily revealed through a joint approach of extraction techniques and high-resolution mass spectrometry analysis. This opens up new scenarios which on the one hand are aimed at obtaining these metabolites, especially the abundant acylated flavonol glycosides, in pure form, and on the other hand at further fractionation strategies for obtaining oak complexes enriched either only in flavonols or only in tannins. This aspect could be relevant, not only for understanding the effect of each polyphenol class, but also for managing the formulation of animal feed products that best exploit the phytochemistry of the oak leaf.

The observation that lipophilic fraction (Qr/2/1) also provided positive outcomes, as it did not affect gas production and fermentation rate, allowed the employment of plant fatty acids and triterpenes to be used to differently modulate rumen fermentation.

Findings herein reported lay the foundation for further studies aimed at utilizing pedunculated oak fractions in optimized formulas as natural modifiers of the rumen fermentation process.

## Figures and Tables

**Table 1:** TOF-MS data of hydrolysable and condensed tannins tentatively identified in oak leaf. RDB = ring double bond equivalent value. Base peak fragments are reported in bold.

**Condensed tannins** * 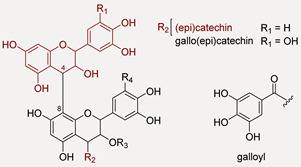 *			**Hydrolysable tannins** * 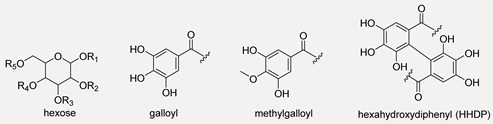 *		
**Condensed Tannins**					
**Peaks**	**R_1_, R_2_ and R_3_**	**(M − H)^−^ Found** ***m*/*z***	**Error** **(ppm)**	**RDB**	**MS/MS Fragment Ions (*m*/*z*)**
**5**	R_1_ = OH; R_2_ = R_3_ = R_4_ = H (I)	593.1318	2.9	18	593.1299; 467.09992; 425.0862; **407.0767**; 381.0983; 339.0861; 289.0706; 245.0804; 205.0496; 177.0187; 125.0244
**6**	R_1_ = R_4_ = OH; R_2_ = R_3_ = H	609.1265	2.5	18	609.1232; 441.0825; **423.0716**; 305.0656; 297.0387; 219.0641; 177.0190; 165.0179; 125.0245
**7**	R_1_ = OH; R_2_ = R_3_ = R_4_ = H (II)	593.1322	3.6	18	425.0854; **407.0759**; 381.0958; 339.0841; 289.0697; 245.0799; 205.0488; 177.0179; 125.0238
**12**	R_1_ = R_2_ = R_3_ = R_4_ = H (I)	577.1349	−0.4	18	577.1368; 451.1026; 425.0861; **407.0769**; 381.0964; 339.0865; 289.0711; 273.0399; 245.0813; 205.0501; 161.0243; 125.0245
**14**	R_1_ = R_2_ = R_3_=R_4_ = H (II)	577.1365	2.3	18	577.1359; 425.0888; 407.0765; 381.0964; 339.0859; **289.0707**; 273.0395; 245.0810; 205.0513; 161.0237; 125.0243
**18**	R_1_ = H = R_3_ = R_4_ = H; R_2_=(epi)catechin (I)	865.1971	−1.7	27	**865.1992**; 847.1872; 739.1667; 713.1516; 695.1415; 677.1283; 587.1193; 577.1353; 575.1204; 525.0818; 451.1037; 449.0848; 425.0876; 413.0874; 407.0763; 405.0610; 341.0652; 289.0706; 287.0550; 243.0291; 161.0242; 125.0243
**19**	R_1_ = H = R_3_ = R_4_ = H; R_2_ = (epi)catechin (II)	865.1971	−1.7	27	**865.1985**; 847.1869; 739.1663; 713.1510; 695.1411; 587.1189; 577.1348; 451.1032; 449.0859; 425.0872; 407.0760; 405.0603; 363.0500; 299.0547; 289.0703; 287.0549; 245.0443; 243.0287; 161.0241; 125.0241
**22**	R_1_ = OH; R_2_ = R_3_ = R_4_ = H (III)	593.1318	2.9	18	593.1314; 509.1305; 467.0954; 425.0898; **407.0754**; 339.0873; 289.0700; 273.0359; 245.0810; 205.0491; 177.0186; 137.0241; 125.0242
**28**	R_1_ = H = R_3_ = R_4_ = H; R_2_ = (epi)catechin (II)	865.1967	−2.1	27	865.1992; 847.1919; 739.1710; 713.1501; 695.1418; 613.1355; 587.1197; 577.1356; 575.1180; 543.0945; 525.0847; 451.1023; 425.0862; 413.0893; **407.0763**, 299.0550; 289.0704; 287.0541; 243.0287; 161.0237; 125.0245
**29**	R_1_ = R_2_ = H = R_4_ = H; R_3_ = galloyl	729.1457	−0.6	23	729.1457; 577.1364; 559.1241; 541.1165; 451.1036; **407.0768**; 381.0968; 299.0548; 289.0709; 287.0550; 269.0434; 245.0453; 169.0132; 125.0238
**31**	R_1_ = R_2_ = R_3_ = R_4_ = H (II)	577.1363	2.0	18	577.1335; 451.1021; 425.0863; 407.0761; 381.0957; **289.0700**; 287.0548; 245.0805; 161.0238; 125.0238.
**Hydrolyzable Tannins**					
**Peaks**	**R_1_, R_2_ and R_3_**	**(M − H)^−^ Found** ***m*/*z***	**Error** **(ppm)**	**RDB**	**MS/MS Fragment Ions (*m*/*z*)**
**9**	R_1_ = R_2_ = HHDP; R_3_ = R_4_ = HHDP; R_5_ = OH	783.0702	2.0	23	783.0693; 481.0625; **300.9975**; 275.0180
**23**	R_1_ = R_2_ = galloyl; R_3_ = R_4_ = HHDP; R_5_ = OH	785.0870	3.4	22	785.0872; 633.0690; 615.0645; 483.0784; 419.0601; **300.9974**; 275.0174; 249.0390
**25**	R_1_ = R_2_ = galloyl; R_3_ = R_4_ = R_5_ = H	467.0843	2.5	11	467.0817; 449.0747; **423.0920**; 374.7814; 315.0709; 313.0545; 241.0324; 169.0127; 152.0116; 125.0252; 124.0151; 109.0290
**27**	R_1_ = galloyl; R_2_ = methylgalloyl; R_3_ = R_4_ = H; R_5_ = OH	497.0951	2.9	11	497.0907; 465.0688; 345.0813; 313.0569; 297.0230; 225.0407; **183.0288**; 169.0127; 124.060
**30**	R_1_ = R_2_ = R_3_ = Gallic acid;R_4_ = H; R_5_ = OH	635.0885	−0.8	16	**465.0670**; 313.0549; 169.0131
**32**	R_1_ = galloyl; R_2_ = R_3_ = HHDP;R_4_ = R_5_ = HHDP	467.0375 [M − 2H]^2−^	nc	19	391.0292; **300.9985**; 275.0195; 169.1045
**37**	R_1_ = R_2_ = R_3_ = galloyl; R_4_ = R_5_ = HHDP	468.0438 [M − 2H]^2−^	nc	27	**300.9974**; 299.9866; 275.0192; 273.0025; 169.0133; 125.0235

**Table 2:** Mono-, di- and acyl-glycosylated flavonols tentatively identified in oak leaf. RDB = ring double bond equivalent value. Base peak fragments are reported in bold.

**Flavonol Skeleton and Main Substituent Residues** * 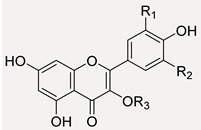 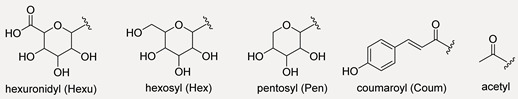 *					
**Kaempferol Derivatives**					
**Peaks**	**R_1_, R_2_ and R_3_**	**[M** **−** **H]^−^ Found** ***m*/*z***	**Error** **(ppm)**	**RDB**	**MS/MS Fragment Ions (*m*/*z*)**
**47**	R_1_ = R_2_ = H; R_3_ = Hex (I)	447.0926	−1.5	12	447.0922; 285.0389; **284.0314**; 255.0285; 227.0339
**48**	R_1_ = R_2_ = H; R_3_ = Hex (II)	447.0919	−3.1	12	447.0947; 285.0397; **284.0320**; 255.0295; 227.0341
**49**	R_1_ = R_2_ = H; R_3_ = Pen-Hex	593.1498	2.9	13	593.1504; 384.9863; 340.9965; **285.0381**; 284.0308; 255.0272
**53**	R_1_ = R_2_ = H; R_3_ = (Acetyl)-Hex (I)	489.1058	4.0	13	489.1043; 285.0393; **284.0312**; 255.0284; 227.0331
**58**	R_1_ = R_2_ = H; R_3_ = (Acetyl)-Hex (I)	489.1035	−0.7	13	489.1020; 285.0394; **284.0315**; 255.0290; 227.0336
**62**	R_1_ = R_2_ = H; R_3_ = *p*-Coum-Hex (I)	593.1323	3.8	18	593.1322; 447.0945; 307.0825; **285.0395**; 284.0317; 255.0288
**63**	R_1_ = R_2_ = R_3_ = H	285.0393	−4.1	11	**285.0391**; 229.0480; 110.9081
**68**	R_1_ = R_2_ = H; R_3_ = *p*-Coum-Hex (II)	593.1336	0.1	18	593.1318; 447.0932; **285.0387**; 284.0309
**72**	R_1_ = R_2_ = H; R_3_ = (Acetyl)-*p*-Coum-Hex	635.1401	−0.6	19	635.1411; 489.1021; **285.0390**; 284.0309; 257.0443; 255.0283
**73**	R_1_ = R_2_ = H; R_3_ = di-*p*-Coum-Hex	739.1654	−2.0	24	739.1664; 593.1335; 575.1186; 453.1176; 307.0787; **285.0388**; 284.0315; 145.0284
**74**	R_1_ = R_2_ = H; R_3_ = *p*-Coum-di-(Acetyl)-Hex (I)	677.1492	−2.9	20	677.1545; 531.1173; **285.0402**; 284.0325; 283.0266
**75**	R_1_ = R_2_ = H; R_3_ = *p*-Coum-di-(Acetyl)-Hex (II)	677.1426	2.1	20	**677.1579**; 617.1397; 531.1201; 285.0406; 284.0326; 283.0230; 255.0296
**78**	R_1_ = R_2_ = H; R_3_ = (Acetyl)-di-*p*-Coum-Hex (I)	781.1759	−1.9	25	781.1770; 635.1395; 617.1257; 575.1170; 495.1284: **285.0390**; 284.0307; 145.0280
**79**	R_1_ = R_2_ = H; R_3_ = (Acetyl)-di-*p*-Coum-Hex (I)	781.1767	−0.8	25	781.1762; 635.1413; 617.1303; 495.1293; **285.0385**; 284.0300; 145.0291
**81**	R_1_ = R_2_ = H; R_3_ = di-(Acetyl)-di-*p*-Coum-Hex (I)	823.1863	−2.0	26	823.1906; 677.1527; 659.1425; **285.0399**; 284.0315; 145.0286
**82**	R_1_ = R_2_ = H; R_3_ = di-(Acetyl)-di-*p*-Coum Hex (II)	823.1865	−1.8	26	823.1874; 677.1511; 659.1409; **285.0388**; 284.0311; 145.0288
**Quercetin Derivatives**					
**Peaks**	**R_1_, R_2_ and R_3_**	**[M − H]^−^ Found** ***m*/*z***	**Error**	**RDB**	**MS/MS Fragment Ions (*m*/*z*)**
**35**	R_1_ = OH; R_2_ = H; R_3_ = di-Hex	625.1431	3.3	13	625.1411; 445.0739; 301.0339; **300.0254**; 271.0230; 178.9979
**38**	R_1_ = OH; R_2_ = H; R_3_ = Pen-Hex (I)	595.1307	0.4	13	595.1329; 301.0348; **300.0274**; 271.0238; 255.0286
**40**	R_1_ = OH; R_2_ = H; R_3_ = Hex (I)	463.0872	−2.2	12	463.0866; 301.0343; **300.0269**; 271.0238; 255.0286
**41**	R_1_ = OH; R_2_ = H; R_3_ = Hexu	477.0661	−2.9	13	**301.0345**; 178.9977; 151.0037
**42**	R_1_ = OH; R_2_ = H; R_3_ = Hex (II)	463.0869	−2.8	12	463.0860; 301.0341; **300.0264**; 271.0235; 255.0283
**43**	R_1_ = OH; R_2_ = H; R_3_ = Pen-Hex (II)	595.1302	−0.4	13	595.1317; 301.0341; **300.0268**; 271.0236; 255.0291
**46**	R_1_ = OH; R_2_ = H; R_3_ = Pen	433.0769	−1.7	12	433.0784; 301.0331; **300.0259**; 271.0229; 255.0277
**54**	R_1_ = OH; R_2_ = R_3_ = H	301.0356	0.7	11	301.0373; 245.0430; 178.9976; **151.0031**; 121.0292; 107.0141
**56**	R_1_ = OH; R_2_ = H; R_3_ = *p*-Coum-Pen-Hex	741.1704	−3.7	10	**741.1679**; 695.3644; 595.1309; 485.2925; 301.0341; 300.0255; 271.0240
**60**	R_1_ = OH; R_2_ = H; R_3_ = *p*-Coum-Hex (I)	609.1254	0.7	18	609.1250; 463.0877; 358.9632; 327.2136; 301.0332; **300.0258**; 271.0235
**61**	R_1_ = OH; R_2_ = H; R_3_ = *p*-Coum-Hex (II)	609.1277	4.5	18	609.1248; 463.0888; 327.2172; 301.0340; **300.0262**; 271.0249
**Isorhamnetin Derivatives**					
**Peaks**	**R_1_, R_2_ and R_3_**	**(M − H)^−^ Found** ***m*/*z***	**Error**	**RDB**	**MS/MS Fragment Ions (*m*/*z*)**
**50**	R_1_ = OCH_3_; R_2_ = H; R_3_ = Pen-Hex	623.1608	−1.5	13	623.1629; **315.0504**; 314.0421; 300.0268; 299.0157
**51**	R_1_ = OCH_3_; R_2_ = H; R_3_ = Hex (I)	477.1039	0.1	12	477.1036; 315.0486; **314.0423**; 300.0264; 299.0174; 285.0389; 271.0235; 257.0442
**52**	R_1_ = OCH_3_; R_2_ = H; R_3_ = Hex (II)	477.1054	3.2	12	477.1032; 315.0488; **314.0417**; 300.0271; 299.0188; 285.0402; 271.0233; 257.0445
**64**	R_1_ = OCH_3_; R_2_ = H; R_3_ = *p*-Coum-Hex	623.1429	3.6	18	623.1394; 477.1020; **315.0492**; 314.0409; 307.0797; 300.0256; 299.0185
**69**	R_1_ = OCH_3_; R_2_ = H; R_3_ = H	315.0495	−4.8	11	315.0544; **300.0273**; 271.0241; 135.0087
**76**	R_1_ = OCH_3_; R_2_ = H; R_3_ = di-*p*-Coum-Hex	769.1789	1.9	24	769.1740; 623.1384; 605.1287; 453.1174; **315.0490**; 314.0444; 307.0795; 300.0247; 145.0284
**Myricetin Derivatives**					
**Peaks**	**R_1_, R_2_ and R_3_**	**(M − H)^−^ Found** ***m*/*z***	**Error**	**RDB**	**MS/MS Fragment Ions (*m*/*z*)**
**33**	R_1_ = OH; R_2_ = OH; R_3_ = Pen-Hex	611.1269	2.5	13	611.1266; 317.0290; **316.0212**; 271.0240
**34**	R_1_ = OH; R_2_ = OH; R_3_ = Hex (I)	479.0826	−1.1	12	479.0824; 317.0299; **316.0212**; 287.0176; 271.0242; 178.9969
**36**	R_1_ = OH; R_2_ = OH; R_3_ = Hex (II)	479.0825	−1.3	12	479.0818; 317.0288; **316.0207**; 287.0170; 271.0231

**Table 3:** (**A**) In vitro cumulative gas production, organic matter degradability and fermentation kinetics parameters of different *Q. robur* extracts. OMD: organic matter degradability; OMCV: cumulative volume of gas related to incubated OM. R_max_: maximum fermentation rate; T_max_: time at which R_max_ occurs. * *p* < 0.05, ** *p* < 0.01 and *** *p* < 0.001. NS: not significant, (**B**) In vitro gas production over time of *Q. robur* Qr/1/1 extract and its fractions Qr/2/1 and Qr/3/2 at 50-mg (●) and 200-mg (●) dose levels and control diet (●).

**(A)**				**(B)**
	**Control Diet**	**Qr/1/1**		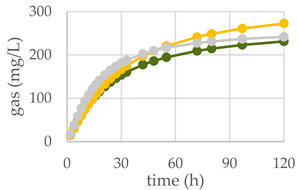
**Parameter**		**50 mg**	**200 mg**	
**OMD (%)**	74.9	65.9 ***	73.8 ^NS^	
**OMCV (mL/g)**	244	220 ***	224 **	
**T_max_ (h)**	3.11	1.77 *	3.86 ^NS^	
**R_max_ (mL/h)**	10.6	8.58 *	8.07 ***	
			
	**Control Diet**	**Qr/2/1**		
		**50 mg**	**200 mg**	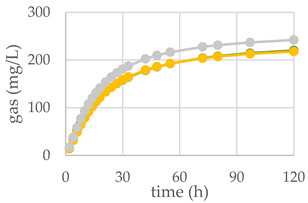
**OMD (%)**	74.9	68.5 ***	72.1 *	
**OMCV (mL/g)**	244	210 ***	184 ***	
**T_max_ (h)**	3.11	1.78 *	3.41 ^NS^	
**R_max_ (mL/h)**	10.6	9.35 ^NS^	8.77 ***	
			
	**Control Diet**	**Qr/3/2**		
		**50 mg**	**200 mg**	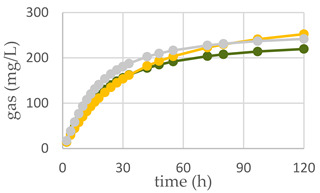
**OMD (%)**	74.9	69.0 ***	65.5 ***	
**OMCV (mL/g)**	244	215 ***	212 **	
**T_max_ (h)**	3.11	3.16 ^NS^	2.12 *	
**R_max_ (mL/h)**	10.6	8.55 *	8.83 ***	
			

**Table 4:** (**A**) Effects of *Q. robur* L. extracts at different doses (50 mg and 200 mg) on fermentation end products after 120 h of incubation. Total VFAs: total volatile fatty acid (acetate + propionate + butyrate + iso-butyrate + valerate + iso-valerate); BCFA: branched-chain fatty acid proportion [(iso-butyrate + iso-valerate)/tVFA] × 100; A/P: acetate/propionate ratio. * *p* < 0.05, ** *p* < 0.01 and *** *p* < 0.001. NS: not significant, (**B**) ViolinPlot of the percentage increase or decrease of end products plotted for different tested dose level (● 50 mg and ■ 200 mg) vs. % in the control diet.

**(A)**				**(B)**
	**Control Diet**	**Qr/1/1**		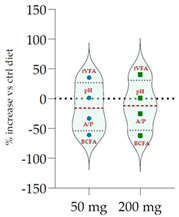
**Parameter**		50 mg	200 mg	
**pH**	6.20	6.29 NS	6.27 NS	
**Total VFA (mmol/L)**	57.20	77.5 ***	80.5 ***	
**BCFA (% VFA)**	7.42	2.90 ***	2.82 ***	
**A/P**	4.27	2.86 ***	3.20 **	
			
	**Control Diet**	**Qr/2/1**		
		50 mg	200 mg	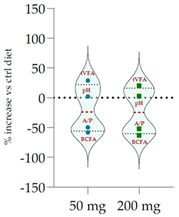
**pH**	6.20	6.33 *	6.37 **	
**Total VFA (mmol/L)**	57.20	73.7 ***	68.9 **	
**BCFA (% VFA)**	7.42	3.11 ***	2.76 ***	
**A/P**	4.27	2.15 ***	2.06 ***	
			
	**Control Diet**	**Qr/3/2**		
		50 mg	200 mg	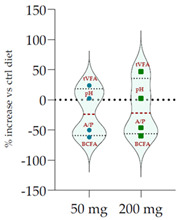
**pH**	6.20	6.35 *	6.34 *	
**Total VFA (mmol/L)**	57.20	70.8 ***	84.0 ***	
**BCFA (% VFA)**	7.42	2.82 ***	3.00 ***	
**A/P**	4.27	2.13 ***	2.30 ***	
			

**Table 5:** Effects of *Q. robur* Qr/1/1 extract and its fractions Qr/2/1 and Qr/3/2 at 50-mg and 200-mg dose levels on fermentation end products after 120 h of incubation. AcA = acetic acid; PrA = propionic acid; ButA = Butyric acid; ValA = valeric acid; iso-ButA = iso-butyric acid; iso-ValA = iso-valeric acid. Along the row * *p* < 0.05, ** *p* < 0.01 and *** *p* < 0.001; NS: not significant; MSE: mean square error. In the lower panel, the percentage increase or decrease of each volatile fatty acid was plotted for different tested dose level (● 50 mg and ● 200 mg) vs. FA% in the control diet.

	**Control Diet**	**Qr/1/1**		**Qr/2/1**		**Qr/3/2**		**MSE**
**(% VFA)**		**50 mg**	**200 mg**	**50 mg**	**200 mg**	**50 mg**	**200 mg**	
**AcA**	64.7	60.0 *	61.8 ^NS^	53.8 ***	53.3 ***	53.1 ***	53.9 ***	0.77
**PrA**	15.1	21.0 ***	19.3 **	25.0 ***	25.9 ***	25.0 ***	23.5 ***	0.31
**ButA**	11.9	15.0 *	14.9 *	17.0 **	17.1 **	18.6 **	18.2 **	0.65
**ValA**	3.98	1.78 ***	1.66 ***	1.91 ***	2.32 ***	1.76 ***	1.96 ***	0.006
**iso-ButA**	0.99	0.85 *	0.85 *	0.96 ^NS^	0.86 *	0.77 **	0.69 **	0.0009
**iso-ValA**	3.25	1.40 ***	1.42 ***	1.38 ***	1.04 ***	1.21 ***	1.82 **	0.03

		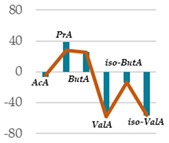		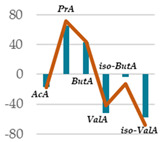		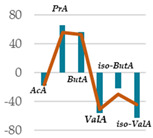		

## Data Availability

Data are within the manuscript and related Supplement Materials.

## Supplementary Materials

The following supporting information can be downloaded at: https://www.mdpi.com/article/10.3390/antiox11122366/s1. Figure S1: In vitro fermentation experimental design. Figure S2: UV/Visible spectra of fractions Qr/2/1 and Qr/3/2. Figure S3: (A) TOF-MS/MS spectrum of compound **27** and (B) putative fragmentation patterns; theoretical mass is reported under each structure. Figure S4: TOF-MS/MS spectra of procyanidins **5** (A), **7** (B) and **22** (C) with [M − H]^−^ ions at *m*/*z* 593.13 and molecular formula C_30_H_26_O_13_. Putative fragmentation patterns are reported with theoretical mass under each structure. Figure S5: TOF-MS/MS spectrum of compound **6**. The structure of main ions, with theoretical mass, is highlighted. Figure S6: TOF-MS/MS spectra of compounds **12** (A), **14** (B) and **31** (C) with [M − H]^−^ ions at *m*/*z* 577.13 and molecular formula C_30_H_26_O_13_. Putative fragmentation patterns are reported with theoretical mass under each structure. Figure S7. TOF-MS/MS spectra of compounds **8** (A), **13** (B) and **16** (C). Putative fragmentation patterns are reported with theoretical mass under each structure. Figure S8: (A) Dendrograms of different volatile fatty acids obtained by treatment at 50 (i.) and 200 mg (ii.); (B) (i.) PCA (% variance on PC1 99.8; on PC2 0.2) of VFAs at 50 mg-dose level; (ii.) PCA (% variance on PC1 99.6; on PC2 0.4) of VFAs 200 of Qr/1/1, Qr/2/1 and Qr/3/2 fractions. Table S1: Compounds tentatively identified in the chestnut Qr/1/1 alcoholic extract and its Qr/2/1 and Qr/3/2 fractions. Rt = retention time; RDB = ring double bond equivalent value. Base peak fragments are reported in bold. Table S2: Values of Pearson’s coefficient correlation, between antiradical (DPPH^●^, ABTS^●+^) activities, reducing activity (PFRAP), total flavonoid content (TFC), total phenol content (TPC), total condensed tannins (TCT) with fermentation parameters at the dose level of 50 mg. tVFA: total volatile fatty acids; AcA = acetic acid; PrA = propionic acid; ButA = Butyric acid; ValA = valeric acid; iso-ButA = iso-butyric acid; iso-ValA = iso-valeric acid; BCFA: branched chain fatty acids (iso-butyrate + iso-valerate/tVFA); A/P=Acetate/Propionate; OMD: organic matter degradability; OMCV: cumulative volume of gas related to incubated organic matter. R_max_: maximum fermentation rate; T_max_: time at which R_max_ occurs. Table S3: Values of Pearson’s coefficient correlation, between antiradical (DPPH^●^, ABTS^●+^) activities, reducing activity (PFRAP), total flavonoid content (TFC), total phenol content (TPC), total condensed tannins (TCT) with fermentation parameters at the dose level of 200 mg. tVFA: total volatile fatty acids; AcA = acetic acid; PrA = propionic acid; ButA = Butyric acid; ValA = valeric acid; iso-ButA = iso-butyric acid; iso-ValA = iso-valeric acid; BCFA: branched chain fatty acids (iso-butyrate + iso-valerate/tVFA); A/P = Acetate/Propionate; OMD: organic matter degradability; OMCV: cumulative volume of gas related to incubated organic matter. R_max_: maximum fermentation rate; T_max_: time at which R_max_ occurs.

## Sample Availability

Samples of the investigated extract and its fractions are available from the authors.
