# Supplementary material for: Antioxidants in Animal Nutrition: UHPLC-ESI-QqTOF Analysis and Effects on In Vitro Rumen Fermentation of Oak Leaf Extracts
Source: Antioxidants (Basel). 2022 Nov 29;11(12):2366. doi: 10.3390/antiox11122366 (PMC9774136; doi:10.3390/antiox11122366)
Supplement: Supplementary file 1 [file antioxidants-11-02366-s001.zip › antioxidants-2060299-supplementary.pdf]

Article

Antioxidants in animal nutrition: UHPLC-ESI-Q*q*TOF analysis and effects on *in vitro* rumen fermentation of oak leaf extracts.

M. Formato,[1] A. Vastolo,[2] S. Piccolella,[1] S. Calabrò,[2] M.I. Cutrignelli,[2] C. Zidorn,[3] S. Pacifico[1]

[1]Department of Environmental, Biological and Pharmaceutical Sciences and Technologies, University of Campania 'Luigi Vanvitelli', Via Vivaldi 43, 81100 Caserta, Italy
[2]Department of Veterinary Medicine and Animal Production, University of Naples Federico II, Via Federico Delpino 1 – 80137, Napoli, Italy
[3]Pharmazeutisches Institut, Abteilung Pharmazeutische Biologie, Christian-Albrechts-Universität zu Kiel, Gutenbergstraße 76, 24118 Kiel, Germany

Supplemental materials

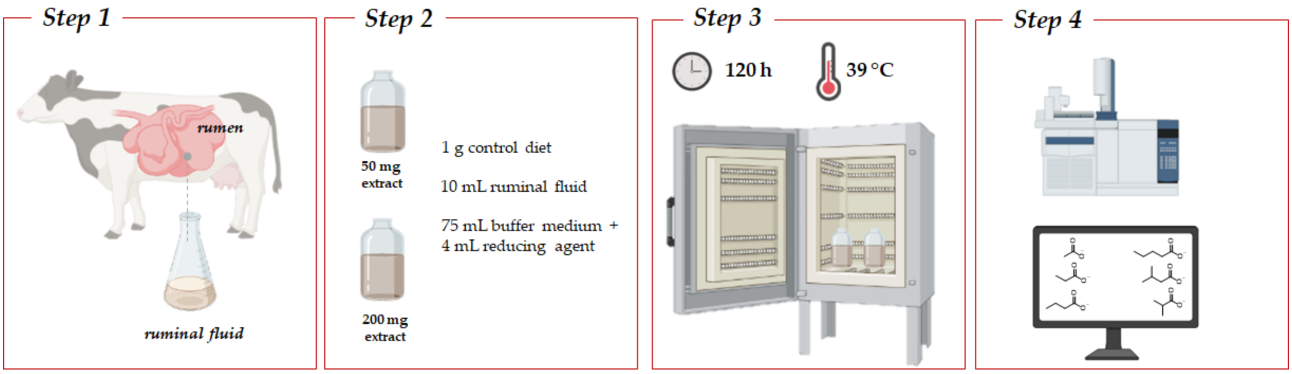

Figure S1. *In vitro* fermentation experimental design

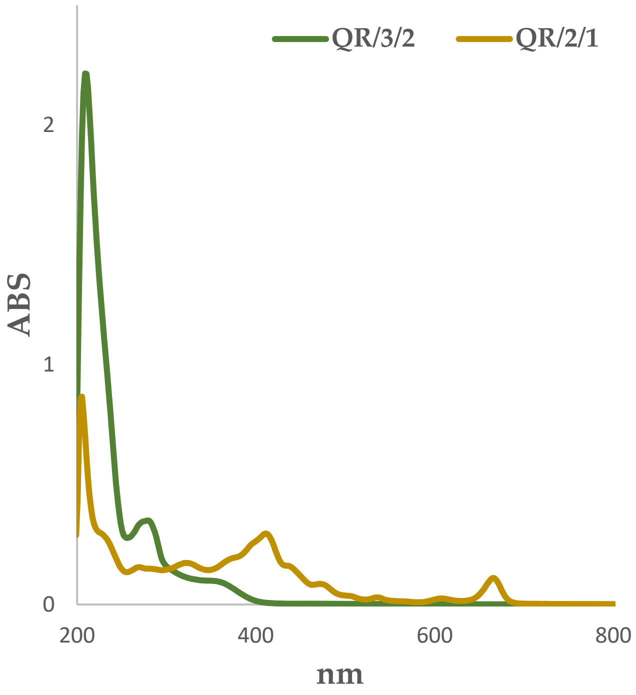

Figure S2. UV/Visible spectra of fractions Qr/2/1 and Qr/3/2.

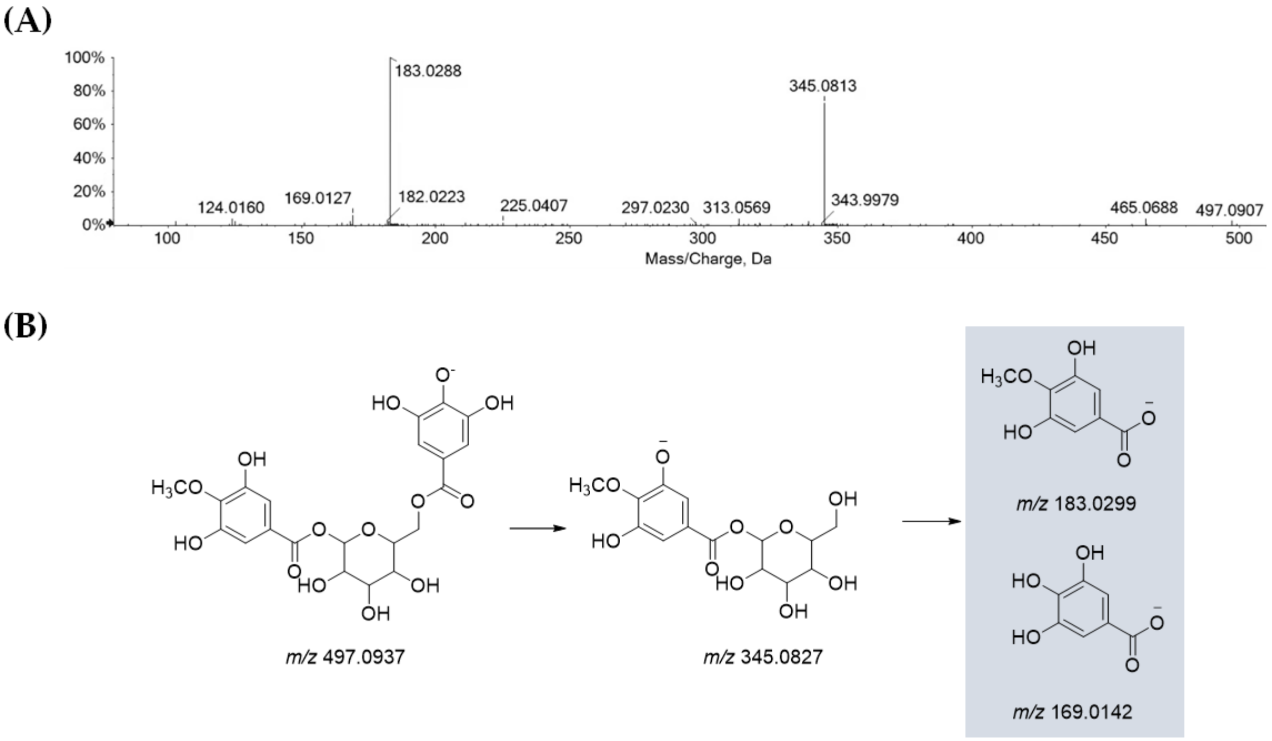

Figure S3. (**A**) TOF-MS/MS spectrum of compound **27** and (**B**) putative fragmentation patterns; theoretical mass is reported under each structure.

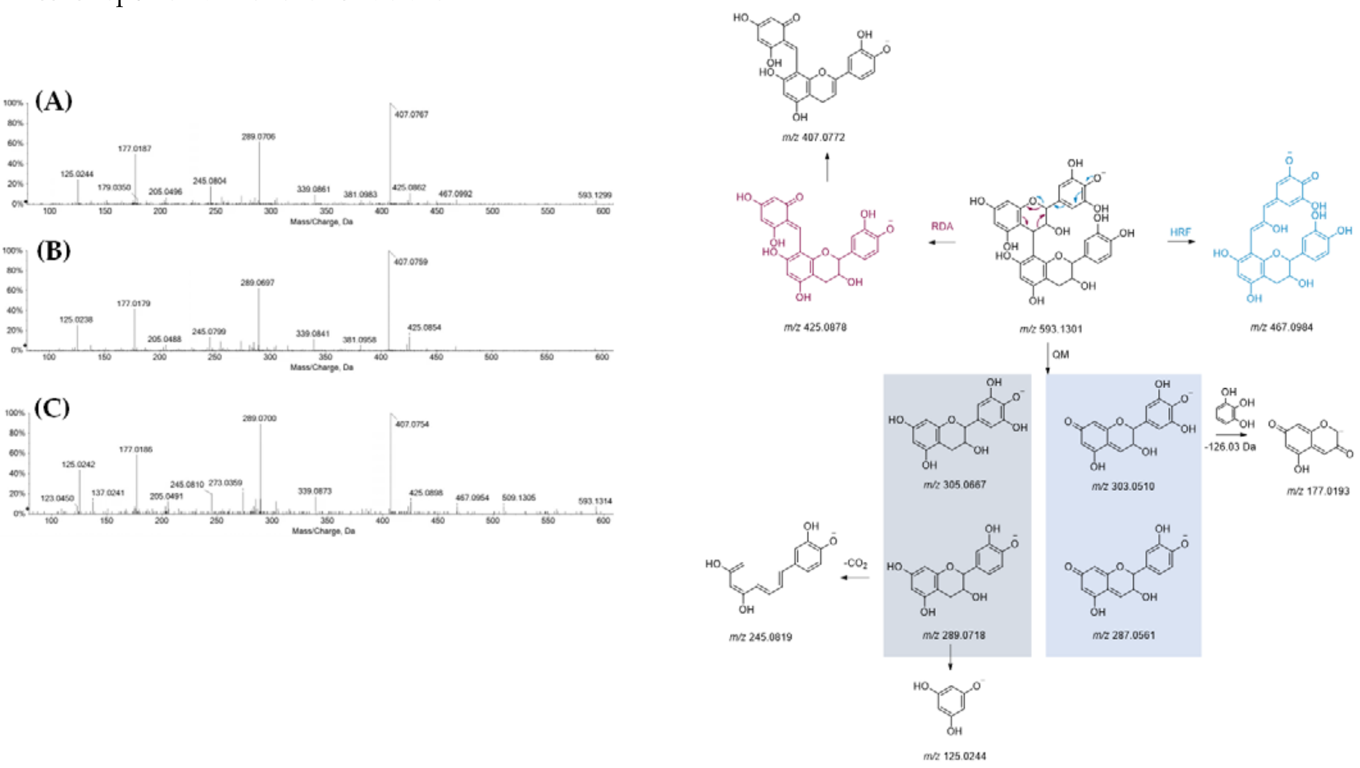

Figure S4. TOF-MS/MS spectra of procyanidins **5** (**A**), **7** (**B**) and **22** (**C**) with [M-H]$^-$ ions at *m/z* 593.13 and molecular formula $C_{30}H_{26}O_{13}$. Putative fragmentation patterns are reported with theoretical mass under each structure.

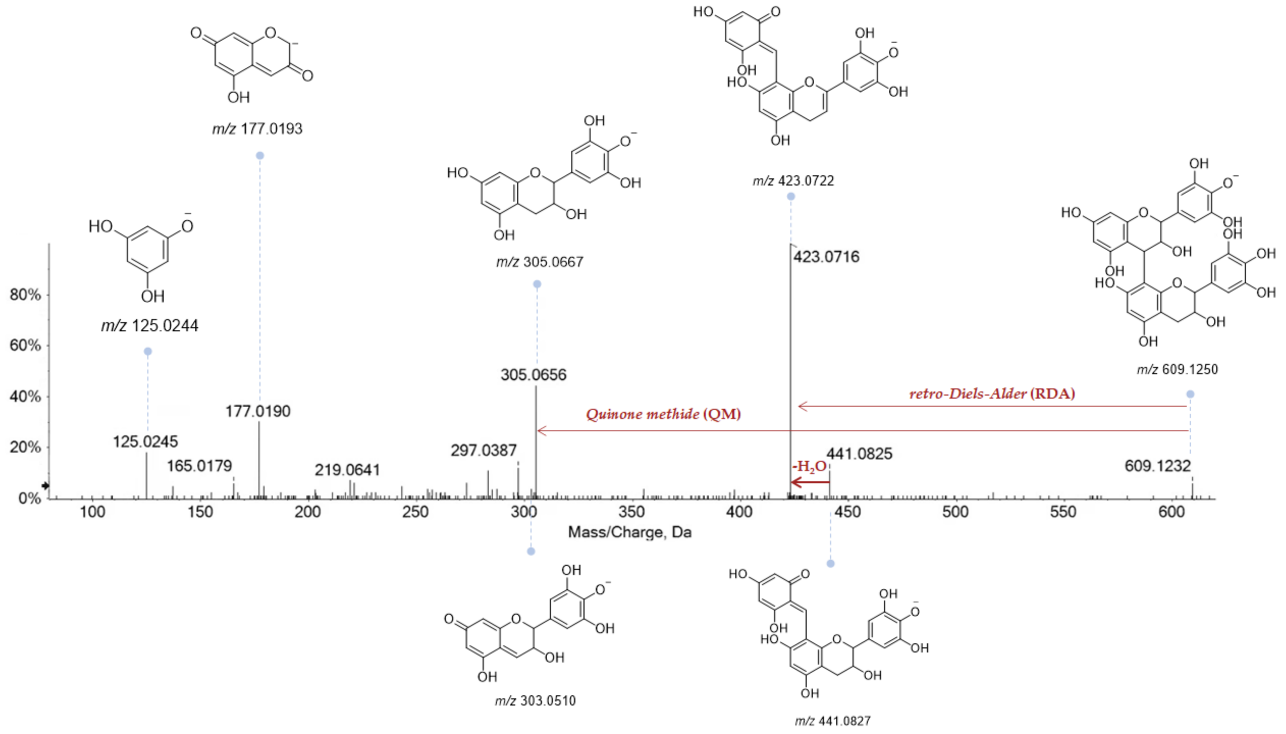

Figure S5. TOF-MS/MS spectrum of compound **6**. The structure of main ions, with theoretical mass, is highlighted.

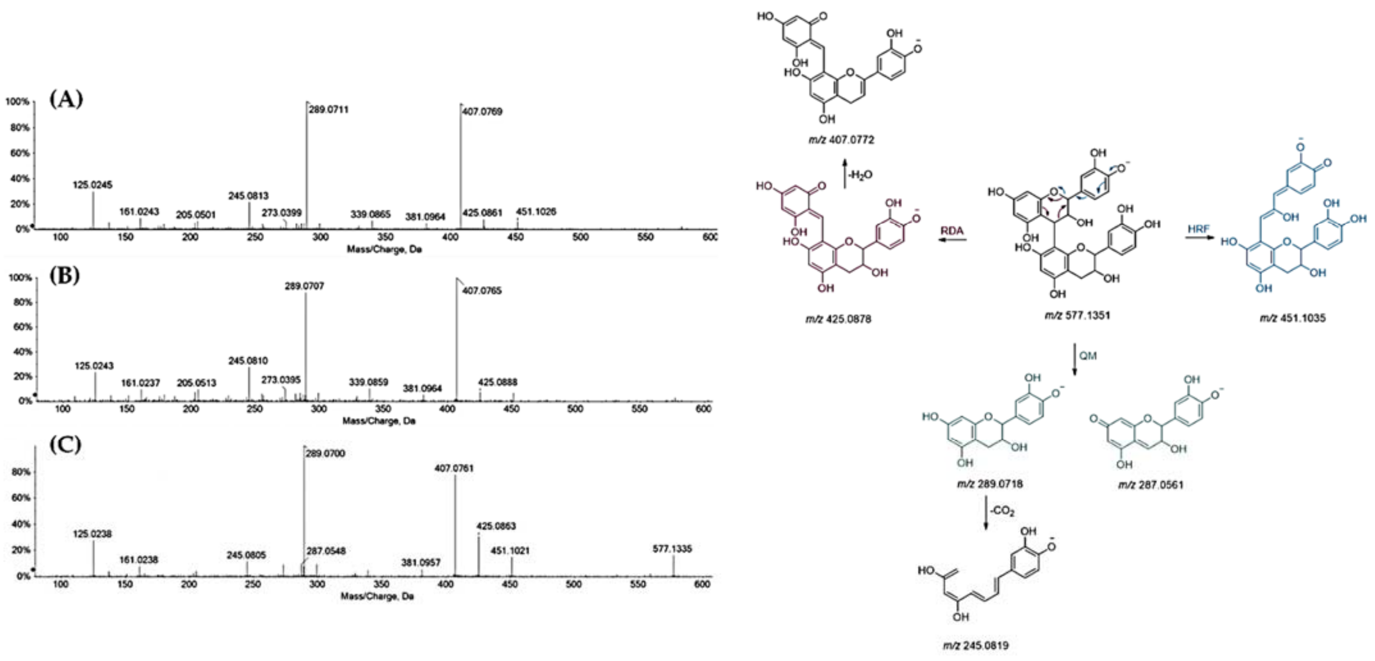

Figure S6. TOF-MS/MS spectra of compounds **12** (**A**), **14** (**B**) and **31** (**C**) with $[M-H]^-$ ions at *m/z* 577.13 and molecular formula $C_{30}H_{26}O_{13}$. Putative fragmentation patterns are reported with theoretical mass under each structure.

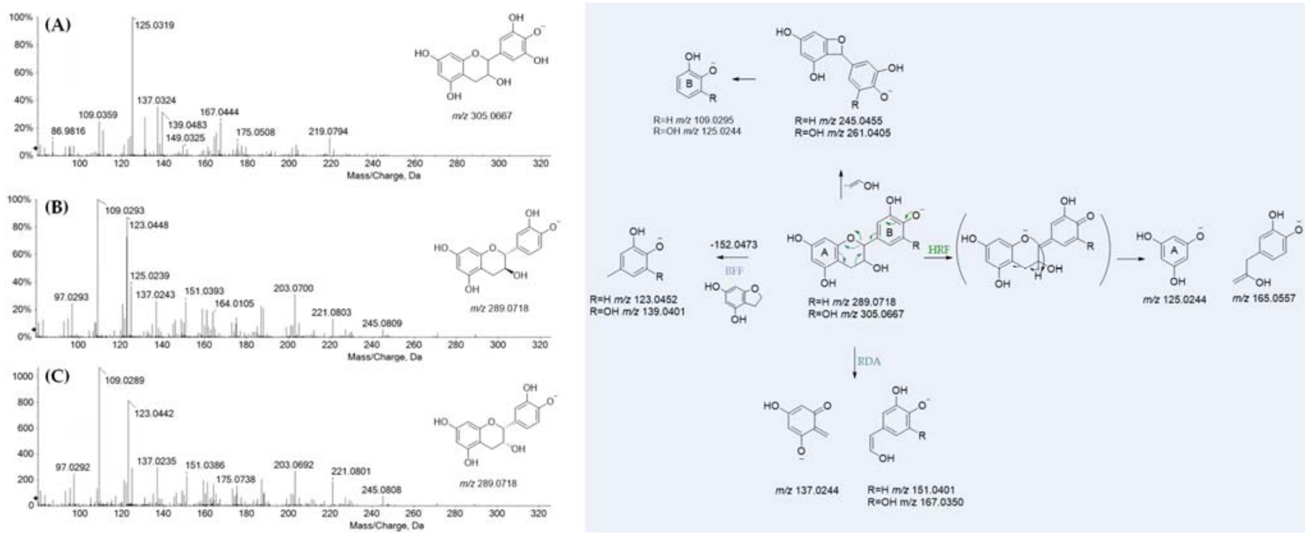

Figure S7. TOF-MS/MS spectra of compounds **8** (**A**)**, 13** (**B**) and **16** (**C**). Putative fragmentation patterns are reported with theoretical mass under each structure.

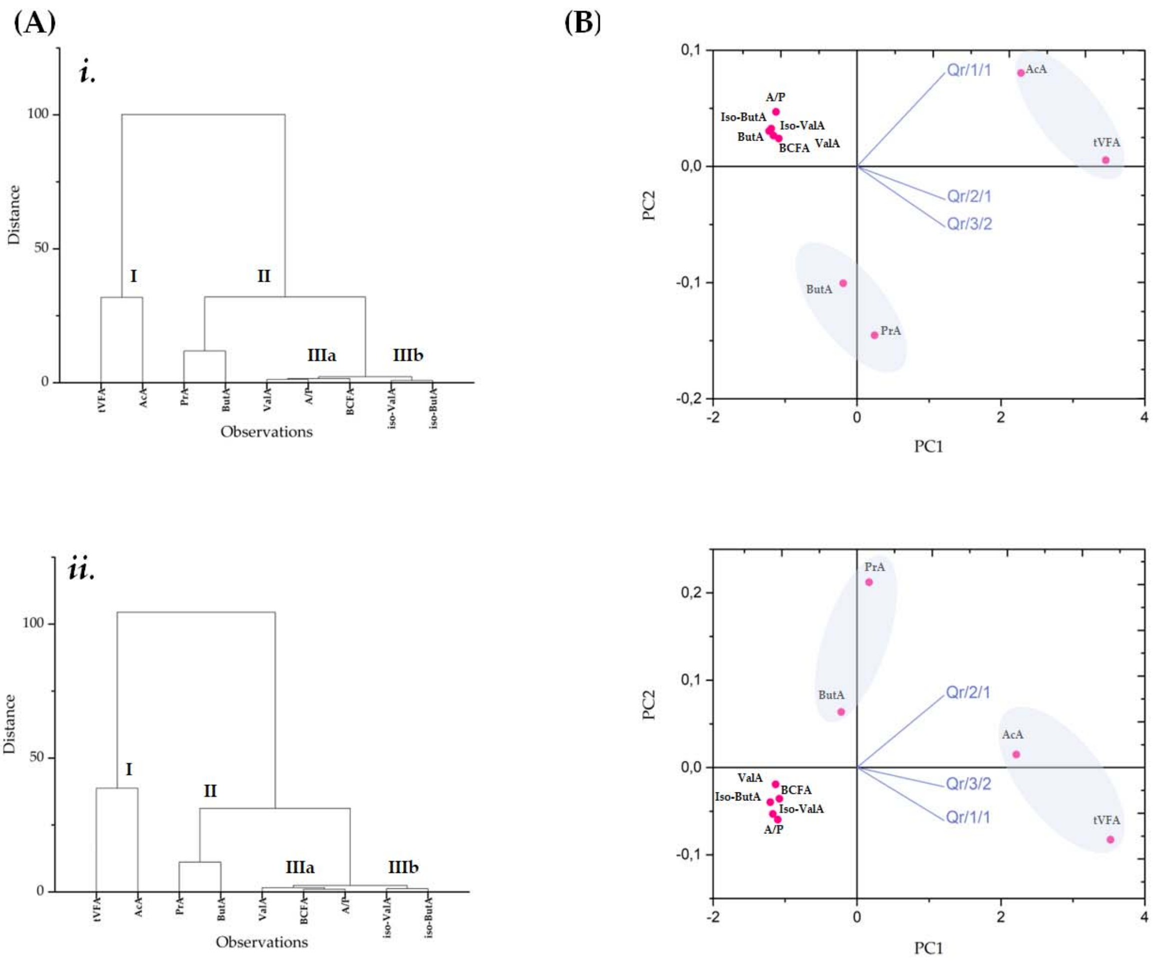

Figure S8. (A) Dendrograms of different volatile fatty acids obtained by treatment at 50 (***i.***) and 200 mg (***ii.***); (**B**) (***i.***) PCA (% variance on PC1 99.8; on PC2 0.2) of VFAs at 50 mg-dose level; (***ii.***) PCA (% variance on PC1 99.6; on PC2 0.4) of VFAs 200 of Qr/1/1, Qr/2/1 and Qr/3/2 fractions.

Table S1. Compounds tentatively identified in the chestnut Qr/1/1 alcoholic extract and its Qr/2/1 and Qr/3/2 fractions. Rt = retention time; RDB = ring double bond equivalent value. Base peak fragments are reported in bold. • Detected.

Peak	Rt (min)	Tentative assignment	Formula	[M-H]⁻ found (*m/z*)	[M-H]⁻ Calc. (*m/z*)	error (ppm)	RDB	MS/MS fragment ions (*m/z*)	Qr/1/1	Qr/2/1	Qr/3/2
**1**	0.343	Quinic acid	$C_7H_{12}O_6$	191.0559	191.0561	-1.1	2	191.0558; 127.0389; 108.0210; 93.0341; **85.0292**	•		•
**2**	0.534	Gallic acid hexoside (I)	$C_{13}H_{16}O_{10}$	331.0676	331.0671	1.6	6	331.0656; 211.0232; **169.0135**; 125.0240; 123.0085	•		•
**3**	0.675	Gallic acid	$C_7H_6O_5$	169.0150	169.0142	4.5	5	**125.0244**; 124.0164; 107.0143; 97.0305	•		•
**4**	0.813	Gallic acid hexoside (II)	$C_{13}H_{16}O_{10}$	331.0657	331.0671	-4.1	6	331.0632; 211.0236; **169.0135**;151.0044; 125.0239; 123.0084	•		•
**5**	1.713	(Epi)gallocatechin-(epi)catechin (procyanidin B-*type*) (I)	$C_{30}H_{26}O_{13}$	593.1318	593.1301	2.9	18	593.1299; 467.09992; 425.0862; **407.0767**; 381.0983; 339.0861; 289.0706; 245.0804; 205.0496; 177.0187; 125.0244	•		•
**6**	1.189	(Epi)gallocatechin-(epi)gallocatechin	$C_{30}H_{26}O_{14}$	609.1265	609.1250	2.5	18	609.1232; 441.0825; **423.0716**; 305.0656; 297.0387; 219.0641; 177.0190; 165.0179; 125.0245	•		•
**7**	1.972	(Epi)gallocatechin-(epi)catechin (procyanidin B-*type*) (II)	$C_{30}H_{26}O_{13}$	593.1322	593.1301	3.6	18	425.0854; **407.0759**; 381.0958; 339.0841; 289.0697; 245.0799; 205.0488; 177.0179; 125.0238	•		•
**8**	2.194	(Epi)gallocatechin	$C_{15}H_{14}O_7$	305.0658	305.0667	-2.9	9	221.0454; 219.0794; 175.0508; 167.0444; 149.0325; 139.0483; 137.0235; **125.0319**; 109.0359; 86.9816	•		•
**9**	2.981	Bis-HHDP-glucose	$C_{34}H_{24}O_{22}$	783.0702	783.0686	2.0	23	783.0693; 481.0625; **300.9975**; 275.0180	•		•
**10**	3.771	*p*-Coumaroyl quinic acid	$C_{16}H_{18}O_8$	337.0915	337.0929	-4.1	8	191.0560; 173.0454; 163.0410; **119.0500**	•		•
**11**	4.161	*p*-Coumaroyl quinic acid	$C_{16}H_{18}O_8$	337.0920	337.0929	-2.6	8	191.0545; 163.0387; **119.0499**	•		•
**12**	4.401	(Epi)catechin-(epi)catechin (I) (procyanidin B-*type*)	$C_{30}H_{26}O_{13}$	577.1349	577.1353	-0.4	18	577.1368; 451.1026; 425.0861; **407.0769**; 381.0964; 339.0865; 289.0711; 273.0399; 245.0813; 205.0501; 161.0243; 125.0245	•		•
**13**	4.479	Catechin	$C_{15}H_{14}O_6$	289.0709	289.0718	-1.9	9	289.0726; 245.0809; 221.0803; 203.0700; 187.0401; 164.0105; 151.0393; 137.0243; 125.0239; 123.0448; **109.0293**; 97.0293	•		•
**14**	4.539	(Epi)catechin-(epi)catechin (II) (procyanidin B-*type*)	$C_{30}H_{26}O_{13}$	577.1365	577.1353	2.3	18	577.1359; 425.0888; 407.0765; 381.0964; 339.0859; **289.0707**; 273.0395; 245.0810; 205.0513; 161.0237; 125.0243	•		•
**15**	5.706	Guaicylglycerol-galloyl hexoside (I)	$C_{23}H_{28}O_{14}$	527.1397	527.1406	-1.8	10	527.1391; 509.1312; **479.1184**; 385.0783; 373.0767; 327.0717; 313.0541; 285.0611; 241.0378; 169.0134; 124.0148	•		•
**16**	5.766	Epicatechin	$C_{15}H_{14}O_6$	289.0705	289.0718	-4.4	9	245.0808; 221.0801; 203.0692; 175.0738; 151.0386; 137.0235; 123.0442; **109.0289**; 97.0292	•		•
**17**	5.349	3-*O*-Feruloyl quinic acid	$C_{17}H_{20}O_9$	367.1027	367.1035	-2.1	8	193.0496; 191.0564; 173.0473; 149.0604; **134.0367;** 117.0340	•		•
**18**	5.986	(Epi)catechin trimer (I)	$C_{45}H_{38}O_{18}$	865.1971	865.1985	-1.7	27	**865.1992**; 847.1872; 739.1667; 713.1516; 695.1415; 677.1283; 587.1193; 577.1353; 575.1204; 525.0818; 451.1037; 449.0848; 425.0876; 413.0874; 407.0763; 405.0610; 341.0652; 289.0706; 287.0550; 243.0291; 161.0242; 125.0243	•		•
**19**	6.246	(Epi)catechin trimer (II)	$C_{45}H_{38}O_{18}$	865.1971	865.1985	-1.7	27	**865.1985**; 847.1869; 739.1663; 713.1510; 695.1411; 587.1189; 577.1348; 451.1032; 449.0859; 425.0872; 407.0760; 405.0603; 363.0500; 299.0547; 289.0703; 287.0549; 245.0443; 243.0287; 161.0241; 125.0241	•		•
**20**	6.259	Eriodictyol 7-O-hexoside	$C_{21}H_{22}O_{11}$	449.1089	449.1089	1.0	11	449.1081; 287.0541; 269.0440**; 259.0601;** 178.9987; 125.0239	•		•

**21**	6.406	Guaicylglycerol-galloyl hexoside (II)	$C_{23}H_{28}O_{14}$	527.1397	527.1406	-1.8	10	527.1403; 509.1291; **479.1179**; 385.0771; 373.0741; 327.0696; 313.0538; 285.0600; 169.0130; 125.0229	•		•
**22**	6.496	(Epi)gallocatechin-(epi)catechin (procyanidin B-*type*) (III)	$C_{30}H_{26}O_{13}$	593.1318	593.1301	2.9	18	593.1314; 509.1305; 467.0954; 425.0898; **407.0754**; 339.0873; 289.0700; 273.0359; 245.0810; 205.0491; 177.0186; 137.0241; 125.0242	•		•
**23**	7.062	Digalloyl-HHDP-glucose	$C_{34}H_{26}O_{22}$	785.0870	785.0843	3.4	22	785.0872; 633.0690; 615.0645; 483.0784; 419.0601; **300.9974**; 275.0174; 249.0390	•		•
**24**	7.106	*p*-Coumaroyl quinic acid	$C_{16}H_{18}O_{8}$	337.0916	337.0929	-3.8	8	**191.0550;** 93.0348	•		•
**25**	7.222	Digalloyl deoxyhexose	$C_{20}H_{20}O_{13}$	467.0843	467.0831	2.5	11	467.0817; 449.0747; **423.0920**; 374.7814; 315.0709; 313.0545; 241.0324; 169.0127; 152.0116; 125.0252; 124.0151; 109.0290	•		•
**26**	7.766	*p*-Coumaroyl quinic acid	$C_{16}H_{18}O_{8}$	337.091	337.0929	-2.9	8	**191.0549**	•		•
**27**	7.972	Galloyl-methylgalloyl hexose	$C_{21}H_{22}O_{14}$	497.0951	497.0937	2.9	11	497.0907; 465.0688; 345.0813; 313.0569; 297.0230; 225.0407; **183.0288**; 169.0127; 124.060	•		•
**28**	8.566	(Epi)catechin trimer (III)	$C_{45}H_{38}O_{18}$	865.1967	865.1985	-2.1	27	865.1992; 847.1919; 739.1710; 713.1501; 695.1418; 613.1355; 587.1197; 577.1356; 575.1180; 543.0945; 525.0847; 451.1023; 425.0862; 413.0893; **407.0763**, 299.0550; 289.0704; 287.0541; 243.0287; 161.0237; 125.0245	•		•
**29**	8.566	(Epi)catechin-(epi)catechin 3-*O*-gallate (procyanidine B-*type*)	$C_{37}H_{30}O_{16}$	729.1457	729.1461	-0.6	23	729.1457; 577.1364; 559.1241; 541.1165; 451.1036; **407.0768**; 381.0968; 299.0548; 289.0709; 287.0550; 269.0434; 245.0453; 169.0132; 125.0238	•		•
**30**	8.566	Trigalloyl hexose	$C_{27}H_{24}O_{18}$	635.0885	635.0890	-0.8	16	**465.0670;** 313.0549; 169.0131	•		•
**31**	8.718	(Epi)catechin-(epi)catechin (III) (procyanidin B-*type*)	$C_{30}H_{26}O_{13}$	577.1363	577.1352	2.0	18	577.1335; 451.1021; 425.0863; 407.0761; 381.0957; **289.0700;** 287.0548; 245.0805; 161.0238; 125.0238.	•		•
**32**	9.017	Galloyl-bis-HHDP-glucose	$C_{41}H_{32}O_{26}$	467.0375 $[M-2H]^{2-}$	935.0796	nc	19	391.0292; **300.9985**; 275.0195; 169.1045	•		•
**33**	9.269	Myricetin pentosyl-hexoside	$C_{26}H_{28}O_{17}$	611.1269	611.1254	2.5	13	611.1266; 317.0290; **316.0212**; 271.0240	•		•
**34**	9.727	Myricetin 3-*O*-hexoside (I)	$C_{21}H_{20}O_{13}$	479.0826	479.0831	-1.1	12	479.0824; 317.0299; **316.0212**; 287.0176; 271.0242; 178.9969	•		•
**35**	9.850	Quercetin di-hexoside	$C_{27}H_{30}O_{17}$	625.1431	625.1410	3.3	13	625.1411; 445.0739; 301.0339; **300.0254**; 271.0230; 178.9979	•		•
**36**	9.988	Myricetin 3-*O*-hexoside (II)	$C_{21}H_{20}O_{13}$	479.0825	479.0831	-1.3	12	479.0818; 317.0288; **316.0207**; 287.0170; 271.0231	•		•
**37**	10.671	Trigalloyl-HHDP-glucose	$C_{41}H_{30}O_{26}$	468.0438 $[M-2H]^{2-}$	937.0953	nc	27	**300.9974**; 299.9866; 275.0192; 273.0025; 169.0133; 125.0235	•		•
**38**	10.791	Quercetin pentosyl-hexoside (I)	$C_{26}H_{28}O_{16}$	595.1307	595.1305	0.4	13	595.1326; 301.0342; **300.0272;** 271.0237; 255.0286	•		•
**39**	11.052	Ellagic acid	$C_{14}H_{6}O_{8}$	300.9996	300.9990	2.0	12	**300.9996**; 299.9910; 283.9973; 229.0146; 185.0241	•		•
**40**	11.212	Quercetin 3-O-hexoside (I)	$C_{21}H_{20}O_{12}$	463.0872	463.0882	-2.2	12	463.0866; 301.0343; **300.0269**; 271.0238; 255.0286	•		•
**41**	11.335	Quercetin hexuronide	$C_{21}H_{18}O_{13}$	477.0661	477.0675	-2.9	13	**301.0345**; 178.9977; 151.0037	•		•
**42**	11.335	Quercetin 3-hexoside (II)	$C_{21}H_{20}O_{12}$	463.0869	463.0882	-2.8	12	463.0860; 301.0341; **300.0264**; 271.0235; 255.0283	•		•
**43**	11.890	Quercetin pentosyl-hexoside (II)	$C_{26}H_{28}O_{16}$	595.1302	595.1305	-0.4	13	595.1317; 301.0341; **300.0268**; 271.0236; 255.0291	•		•
**44**	11.938	Neolignan-9′-*O*- rhamnoside (I)	$C_{25}H_{34}O_{11}$	509.2049	509.2028	4.1	9	473.1841; 367.1404; **313.1288;** 179.0709; 161.0604; 149.0602	•		•
**45**	12.025	Neolignan-9′-*O*- rhamnoside (II)	$C_{25}H_{34}O_{11}$	509.2047	509.2028	3.7	9	473.1815; 367.1393; **313.1287;** 179.0713; 161.0611; 149.0607	•		•
**46**	12.310	Quercetin 3-*O*-pentoside	$C_{20}H_{18}O_{11}$	433.0769	433.0776	-1.7	12	433.0784; 301.0331; **300.0259**; 271.0229; 255.0277	•		•
**47**	12.397	Kaempferol 3-*O*-hexoside (I)	$C_{21}H_{22}O_{11}$	447.0926	447.0933	-1.5	12	447.0922; 285.0389; **284.0314**; 255.0285; 227.0339	•		•
**48**	12.911	Kaempferol 3-*O*-hexoside (II)	$C_{21}H_{22}O_{11}$	447.0919	447.0933	-3.1	12	447.0947; 285.0397; **284.0320**; 255.0295; 227.0341	•		•
**49**	12.911	Kaempferol pentosyl-hexoside	$C_{27}H_{30}O_{15}$	593.1498	593.1512	2.9	13	593.1504; 384.9863; 340.9965; **285.0381**; 284.0308; 255.0272	•		•

**50**	13.328	Isorhamnetin pentosyl-hexoside	$C_{28}H_{32}O_{16}$	623.1608	623.1618	-1.5	13	623.1629; **315.0504**; 314.0421; 300.0268; 299.0157	•		•
**51**	13.368	Isorhamnetin hexoside (I)	$C_{22}H_{22}O_{12}$	477.1039	477.1039	0.1	12	477.1036; 315.0486; **314.0423**; 300.0264; 299.0174; 285.0389; 271.0235; 257.0442	•		•
**52**	14.196	Isorhamnetin hexoside (II)	$C_{22}H_{22}O_{12}$	477.1054	477.1039	3.2	12	477.1032; 315.0488; **314.0417**; 300.0271; 299.0188; 285.0402; 271.0233; 257.0445	•		•
**53**	14.436	Kaempferol (acetyl)-hexoside (I)	$C_{23}H_{22}O_{12}$	489.1058	489.1039	4.0	13	489.1043; 285.0393; **284.0312**; 255.0284; 227.0331	•	•	•
**54**	14.569	Quercetin	$C_{15}H_{10}O_{7}$	301.0356	301.0354	0.7	11	301.0373; 245.0430; 178.9976; **151.0031;** 121.0292; 107.0141	•	•	•
**55**	14.714	Hydroxy-dihydrojasmonic acid hexoside (I)	$C_{18}H_{30}O_{9}$	389.1808	389.1817	-2.3	4	227.1270;197.1527; **183.1375**; 165.1266	•	•	•
**56**	14.783	Quercetin *p*-coumaroyl-pentosylhexoside	$C_{28}H_{38}O_{23}$	741.1704	741.1731	-3.7	10	**741.1679**; 695.3644; 595.1309; 485.2925; 301.0341; 300.0255; 271.0240	•		•
**57**	14.963	Hydroxy-dihydrojasmonic acid hexoside (II)	$C_{18}H_{30}O_{9}$	389.1825	389.1817	2.0	4	251.1266; 227.1290;197.1538; **183.1385**; 165.1279	•	•	•
**58**	15.075	Kaempferol (acetyl)-hexoside (II)	$C_{23}H_{22}O_{12}$	489.1035	489.1039	-0.7	13	489.1020; 285.0394; **284.0315**; 255.0290; 227.0336	•	•	•
**59**	15.104	Pentacyclic triterpene hexoside	$C_{36}H_{58}O_{11}$	711.3993<br>$[M+FA]^-$	665.3906	nc	8	711.3988; 665.3944; **503.3395**	•		•
**60**	15.155	Quercetin *p*-coumaroyl hexoside (I)	$C_{30}H_{26}O_{14}$	609.1254	609.1250	0.7	18	609.1250; 463.0877; 358.9632; 327.2136; 301.0332; **300.0258**; 271.0235	•		•
**61**	15.304	Quercetin *p*-coumaroyl hexoside (II)	$C_{30}H_{26}O_{14}$	609.1277	609.1250	4.5	18	609.1248; 463.0888; 327.2172; 301.0340; **300.0262**; 271.0249	•		•
**62**	15.884	Kaempferol *p*-coumaroyl hexoside (I)	$C_{30}H_{26}O_{13}$	593.1323	593.1301	3.8	18	593.1322; 447.0945; 307.0825; **285.0395**; 284.0317; 255.0288	•		•
**63**	16.015	Kaempferol	$C_{15}H_{10}O_{6}$	285.0393	285.0405	-4.1	11	**285.0391**; 229.0480; 110.9081	•	•	•
**64**	16.124	Isorhamnetin *p*-coumaroyl hexoside	$C_{31}H_{28}O_{14}$	623.1429	623.1406	3.6	18	623.1394; 477.1020; **315.0492**; 314.0409; 307.0797; 300.0256; 299.0185	•	•	•
**65**	16.124	Bartogenic acid hexoside (I)	$C_{36}H_{56}O_{12}$	679.3720<br>725.3748<br>$[M+FA]^-$	679.3699	3.1	9	**679.3692**; 559.3264; 517.3149; 499.3042; 455.3157	•		•
**66**	16.262	Bartogenic acid hexoside (II)	$C_{36}H_{56}O_{12}$	679.3718<br>725.3748<br>$[M+FA]^-$	679.3699	2.8	9	**679.3704;** 559.3272**;** 517.3174; 455.3162; 437.3046	•		•
**67**	16.355	Dodecanedioic acid	$C_{12}H_{20}O_{4}$	227.1287	227.1289	-0.8	3	183.1376; **136.9225**	•	•	•
**68**	16.514	Kaempferol *p*-coumaroyl hexoside (II)	$C_{30}H_{26}O_{13}$	593.1336	593.1301	0.1	18	593.1318; 447.0932; **285.0387**; 284.0309	•		•
**69**	16.395	Isorhamnetin	$C_{16}H_{12}O_{7}$	315.0495	315.0510	-4.8	11	315.0544; **300.0273**; 271.0241; 135.0087	•	•	•
**70**	16.574	9,12,13-trihydroxy-10,15 octadecadienoic acid	$C_{18}H_{32}O_{5}$	327.2172	327.2177	-1.5	3	327.2160; 291.1951; 229.1432; **211.1325**; 183.1391; 171.1018	•	•	•
**71**	17.314	9,12,13-trihydroxy-10-octadecenoic acid	$C_{18}H_{34}O_{5}$	329.2320	329.2333	-4.1	2	329.2329; 229.1440; **211.1339**; 171.1039	•	•	•
**72**	17.314	Kaempferol (acetyl)-*p*-coumaroyl-hexoside	$C_{32}H_{28}O_{14}$	635.1401	635.1406	-0.6	19	635.1411; 489.1021; **285.0390**; 284.0309; 257.0443; 255.0283	•	•	•
**73**	18.531	Kaempferol di-*p*-coumaroyl hexoside	$C_{39}H_{32}O_{15}$	739.1654	739.1668	-2.0	24	739.1664; 593.1335; 575.1186; 453.1176; 307.0787; **285.0388**; 284.0315; 145.0284	•	•	•

**74**	18.671	Kaempferol *p*-coumaroyl-di-(acetyl)-hexoside (I)	$C_{34}H_{30}O_{15}$	677.1492	677.1512	-2.9	20	677.1545; 531.1173; **285.0402**; 284.0325; 283.0266	•	•	•
**75**	18.910	Kaempferol *p*-coumaroyl-di-(acetyl)-hexoside (II)	$C_{34}H_{30}O_{15}$	677.1426	677.1512	2.1	20	**677.1579;** 617.1397; 531.1201; 285.0406; 284.0326; 283.0230; 255.0296	•	•	•
**76**	18.684	Isorhamentin di-*p*-coumaroyl hexoside	$C_{40}H_{34}O_{16}$	769.1789	769.1774	1.9	24	769.1740; 623.1384; 605.1287; 453.1174; **315.0490;** 314.0444; 307.0795; 300.0247; 145.0284	•	•	•
**77**	18.951	Bartogenic acid	$C_{30}H_{56}O_7$	517.3171	517.3171	0.0	8	**517.3173**; 499.3041; 471.3113; 455.3161; 437.3051	•	•	•
**78**	19.531	Kaempferol (acetyl)-di-*p*-coumaroyl-hexoside (I)	$C_{41}H_{34}O_{16}$	781.1759	781.1774	-1.9	25	781.1770; 635.1395; 617.1257; 575.1170; 495.1284: **285.0390**; 284.0307; 145.0280	•	•	•
**79**	19.731	Kaempferol (acetyl)-di-*p*-coumaroyl-hexoside (II)	$C_{41}H_{34}O_{16}$	781.1767	781.1774	-0.8	25	781.1762; 635.1413; 617.1303; 495.1293; **285.0385**; 284.0300; 145.0291	•	•	•
**80**	20.324	Pentacyclic triterpene	$C_{30}H_{48}O_5$	487.3443 533.3498 [M-H]-	487.3429	2.9	7	**487.3436;** 469.3281; 409.3076	•	•	•
**81**	20.790	Kaempferol di-(acetyl)-di-*p*-coumaroyl-hexoside (I)	$C_{43}H_{36}O_{17}$	823.1863	823.1880	-2.0	26	823.1906; 677.1527; 659.1425; **285.0399**; 284.0315; 145.0286	•	•	•
**82**	20.928	Kaempferol di-(acetyl)-di-*p*-coumaroyl-hexoside (II)	$C_{43}H_{36}O_{17}$	823.1865	823.1880	-1.8	26	823.1874; 677.1511; 659.1409; **285.0388**; 284.0311; 145.0288	•	•	•
**83**	21.346	DGMG (18:3)	$C_{33}H_{56}O_{14}$	721.3641 [M+FA]-	675.3597	nc	6	675.3636; 415.1478; **397.1373**; 277.2176; 235.0825; 89.0239	•	•	•
**84**	21.485	9-hydroxy-10,12,15-octadecatrienoic acid	$C_{18}H_{30}O_3$	293.2113	293.2122	-3.1	4	293.2068; 275.1985; **171.1017**; 121.1034	•	•	
**85**	22.168	l-PA (18:3)	$C_{21}H_{37}O_7P$	431.2198	431.2204	-1.4	4	431.2232; 277.2185; **152.9961**	•	•	•
**86**	22.308	MGMG (18:3)	$C_{27}H_{46}O_9$	[M+FA]- 559.3127	513.3069	nc		**277.2163**; 253.0910	•	•	•
**87**	22.886	Maslinic/Corsolic acid	$C_{30}H_{48}O_4$	471.3480	471.3480	-0.4	7	**471.3469**	•	•	
**88**	23.056	l-PA (18:2)	$C_{21}H_{39}O_7P$	433.2351	433.2361	-2.2	3	279.2308; **152.9950**	•	•	•
**89**	23.520	Oleoyl-diglycerol-phosphate	$C_{24}H_{47}O_9P$	509.2879	509.2885	-1.2	2	509.2884; **281.2475**; 152.9952	•	•	•
**90**	25.142	Linolenic acid	$C_{18}H_{30}O_2$	277.2179	277.2173	-2.1	4	**277.2154**	•	•	
**91**	25.518	Ursolic/Oleanolic acid	$C_{30}H_{48}O_3$	455.3546	455.3531	3.4	7	**455.3546**	•	•	
**92**	26.195	Linoleic acid	$C_{18}H_{32}O_2$	279.2332	279.2330	0.9	3	**279.2360**	•	•	
**93**	26.420	DGDG (18:3-O/18:3)	$C_{51}H_{84}O_{16}$	997.5744 [M+FA]-	951.5687	nc	10	951.5798; 691.3619; 673.3508; 657.3560; 415.1478; 397.1376; 379.1268; **293.2133**; 277.2180	•	•	
**94**	27.353	Oleic acid	$C_{18}H_{34}O_2$	281.2488	281.2486	0.7	2	**281.2506**	•	•	
**95**	29.160	DGDG (18:3/18:3)	$C_{51}H_{84}O_{15}$	981.5795 [M+FA]-	935.5737	nc	10	935.5824; 675.3652; 657.3551; 415.1473; 397.1370; 379.1261; **277.2181**	•	•	•
**96**	31.019	Eicosyl *p*-coumarate	$C_{29}H_{48}O_3$	443.3547	443.3532	3.7	6	**443.3553**; 163.0388; 145.0287; 119.0487	•	•	
**97**	31.179	Docosyl caffeate	$C_{31}H_{52}O_4$	487.3811	487.3793	3.7	6	**487.3824;** 179.0394; 161.0241; 134.0374	•	•	

Table S2. Values of Pearson's coefficient correlation, between antiradical (DPPH•, ABTS•+) activities, reducing activity (PFRAP), total flavonoid content (TFC), total phenol content (TPC), total condensed tannins (TCT) with fermentation parameters at the dose level of 50 mg. tVFA: total volatile fatty acids; AcA = acetic acid; PrA = propionic acid; ButA = Butyric acid; ValA = valeric acid; iso-ButA = iso-butyric acid; iso-ValA = iso-valeric acid; BCFA: branched chain fatty acids (iso-butyrate + iso-valerate/tVFA); A/P=Acetate/Propionate; OMD: organic matter degradability; OMCV: cumulative volume of gas related to incubated organic matter. R_{max}: maximum fermentation rate; T_{max}: time at which R_{max} occurs.

	TFC	*TPC*	*TCT*	*ABTS*	*DPPH*	*FRAP*	*pH*	*tVFA*	*AcA*	*PrA*	*ButA*	*ValA*	*iso-ButA*	*iso-ValA*	*BCFA*	*A/P*	*OMD*	*OMCV*	R_{max}	T_{max}
TFC	1,000																			
TPC	0,979	1,000																		
TCT	0,991	0,997	1,000																	
ABTS	0,981	1,000	0,998	1,000																
DPPH	0,995	0,995	0,999	0,996	1,000															
FRAP	0,998	0,964	0,980	0,966	0,986	1,000														
pH	0,560	0,379	0,445	0,387	0,473	0,613	1,000													
tVFA	-0,649	-0,481	-0,543	-0,489	-0,569	-0,698	-0,994	1,000												
AcA	-0,346	-0,147	-0,218	-0,156	-0,249	-0,407	-0,971	0,938	1,000											
PrA	0,258	0,055	0,127	0,064	0,158	0,321	0,945	-0,902	-0,996	1,000										
ButA	0,660	0,493	0,554	0,500	0,580	0,707	0,992	-1,000	-0,933	0,896	1,000									
ValA	-0,789	-0,898	-0,864	-0,894	-0,848	-0,747	0,067	0,046	-0,303	0,390	-0,059	1,000								
Iso-ButA	-0,939	-0,989	-0,976	-0,988	-0,969	-0,914	-0,240	0,348	0,001	0,091	-0,360	0,952	1,000							
Iso-ValA	-0,936	-0,845	-0,881	-0,850	-0,896	-0,957	-0,815	0,875	0,653	-0,581	-0,882	0,523	0,758	1,000						
BCFA	-0,871	-0,953	-0,928	-0,950	-0,916	-0,837	-0,080	0,192	-0,160	0,251	-0,205	0,989	0,987	0,643	1,000					
A/P	-0,281	-0,079	-0,151	-0,088	-0,182	-0,343	-0,953	0,912	0,998	-1,000	-0,907	-0,368	-0,067	0,600	-0,227	1,000				
OMD	0,400	0,205	0,274	0,213	0,305	0,459	0,983	-0,957	-0,998	0,989	0,953	0,247	-0,060	-0,696	0,102	-0,992	1,000			
OMCV	0,260	0,451	0,386	0,443	0,357	0,196	-0,655	0,565	0,816	-0,866	-0,554	-0,798	-0,577	0,096	-0,701	0,854	-0,781	1,000		
R_{max}	-0,731	-0,855	-0,815	-0,850	-0,796	-0,685	0,156	-0,044	-0,388	0,471	0,031	0,996	0,921	0,445	0,972	-0,450	0,333	-0,849	1,000	
T_{max}	0,964	0,890	0,920	0,894	0,932	0,979	0,760	-0,828	-0,583	0,505	0,836	-0,598	-0,813	-0,996	-0,709	-0,526	0,629	-0,006	-0,523	1,000

Table S3. Values of Pearson's coefficient correlation, between antiradical (DPPH•, ABTS•+) activities, reducing activity (PFRAP), total flavonoid content (TFC), total phenol content (TPC), total condensed tannins (TCT) with fermentation parameters at the dose level of 200 mg. tVFA: total volatile fatty acids; AcA = acetic acid; PrA = propionic acid; ButA = Butyric acid; ValA = valeric acid; iso-ButA = iso-butyric acid; iso-ValA = iso-valeric acid; BCFA: branched chain fatty acids (iso-butyrate + iso-valerate/tVFA); A/P=Acetate/Propionate; OMD: organic matter degradability; OMCV: cumulative volume of gas related to incubated organic matter. R_{max}: maximum fermentation rate; T_{max}: time at which R_{max} occurs.

	TFC	*TPC*	*TCT*	*ABTS*	*DPPH*	*FRAP*	*pH*	*tVFA*	*AcA*	*PrA*	*ButA*	*ValA*	*iso-ButA*	*iso-ValA*	*BCFA*	*A/P*	*OMD*	*OMCV*	R_{max}	T_{max}
TFC	1,000																			
TPC	0,979	1,000																		
TCT	0,991	0,997	1,000																	
ABTS	0,981	1,000	0,998	1,000																
DPPH	0,995	0,995	0,999	0,996	1,000															
FRAP	0,998	0,964	0,980	0,966	0,986	1,000														
pH	-0,036	-0,239	-0,169	-0,230	-0,137	0,030	1,000													
tVFA	0,847	0,937	0,910	0,934	0,896	0,810	-0,562	1,000												
AcA	-0,196	0,008	-0,064	-0,001	-0,096	-0,260	-0,973	0,356	1,000											
PrA	-0,107	-0,307	-0,238	-0,299	-0,207	-0,041	0,997	-0,619	-0,954	1,000										
ButA	0,560	0,379	0,445	0,387	0,473	0,613	0,808	0,033	-0,922	0,764	1,000									
ValA	-0,310	-0,497	-0,434	-0,490	-0,405	-0,247	0,961	-0,768	-0,871	0,978	0,614	1,000								
Iso-ButA	-0,978	-0,915	-0,941	-0,918	-0,952	-0,990	-0,174	-0,717	0,397	-0,104	-0,721	0,105	1,000							
Iso-ValA	0,970	0,999	0,994	0,999	0,990	0,952	-0,278	0,951	0,048	-0,345	0,341	-0,532	-0,898	1,000						
BCFA	1,000	0,975	0,988	0,977	0,993	0,999	-0,016	0,836	-0,216	-0,086	0,577	-0,291	-0,982	0,965	1,000					
A/P	-0,060	0,145	0,074	0,136	0,042	-0,125	-0,995	0,481	0,991	-0,986	-0,861	-0,931	0,267	0,185	-0,080	1,000				
OMD	-0,897	-0,788	-0,830	-0,793	-0,847	-0,924	-0,410	-0,524	0,609	-0,344	-0,869	-0,142	0,970	-0,762	-0,906	0,495	1,000			
OMCV	0,471	0,640	0,584	0,634	0,558	0,412	-0,899	0,868	0,773	-0,927	-0,468	-0,985	-0,276	0,671	0,452	0,853	-0,032	1,000		
R_{max}	0,326	0,126	0,197	0,135	0,228	0,387	0,933	-0,227	-0,991	0,905	0,966	0,798	-0,516	0,086	0,345	-0,963	-0,710	-0,681	1,000	
T_{max}	-0,870	-0,752	-0,797	-0,758	-0,816	-0,901	-0,461	-0,475	0,653	-0,397	-0,895	-0,198	0,954	-0,724	-0,880	0,543	0,998	0,025	-0,749	1,000
